# Building schemas through the lived experiences of an African American roller coaster engineer: Interdisciplinary analyses of students’ dialogues in a physics unit

**DOI:** 10.3389/fsoc.2026.1859066

**Published:** 2026-10-09

**Authors:** Catherine L. Quinlan, Elizabeth R. Rollins, Rasheda Likely, Delphia Smith, TaVshea Smith

**Affiliations:** 1 Curriculum and Instruction, North Carolina Central University, Durham, NC, United States; 2 Elementary and Early Childhood Education, Kennesaw State University, Kennesaw, GA, United States; 3 Curriulum and Instruction, Howard University, Washington, DC, United States

**Keywords:** African Americans, Black representation, roller coaster physics, STEM curricula, schema theory, socioemotional learning, funds of knowledge

## Abstract

This research is part of a larger goal to capture the heritage, lived experiences, and scientific and cultural capital of Black people in the United States in the science curricula. This research examines the impact of a Science, Technology, Engineering, and Mathematics (STEM) physics curriculum created for the K-12 classroom, which began with the lived experiences and narratives of an African American engineer. Multimedia and other curriculum products were developed and implemented using best practices in science education pedagogy. Participants were Black homeschoolers ages 3 to 14 and their parents located in the Mid-Atlantic region of the United States. Implementation was virtual, with Black homeschooled children meeting weekly on Zoom for 1 hour for a total of 8 weeks. This study used a pragmatic approach that combined the disciplinary expertise of multiple researchers to identify the best frameworks for capturing the emergent themes and essence of the interactions from video recordings and transcripts. Schema theory and Funds of Knowledge served as frameworks to explore and explain the large volume of video dialogues and transcripts emerging from this implementation. The study revealed themes and patterns which support best practices in content creation that centers Black narratives and lived experiences in the science curricula. The research has important implications for creating curricula that center cultural and heritage knowledge in meaningful ways in the science and STEM curricula using interdisciplinary and multidisciplinary approaches.

## Introduction

This research highlights the benefits of using the lived experiences and narratives of Black people as integral to learning and driving the science curricula and content within a Black homeschooling community. Researchers noted that one failing of curricula or research that purported to be culturally relevant or sustaining was that understandings were not derived from the people in that culture (Ladson-Billings, 2021; Ladson-Billings et al., 2024). This research focuses on advancing understandings about curricula designed to tap into and promote the motivation, interests, and community and familial assets Black children bring with them. This study does not focus on the curriculum design or development; instead, it asks questions about the benefits of the curriculum and what we can learn from implementing it within a virtual homeschooling learning space. While this work focused on implementation outcomes, the significance was in the curriculum treatment used. The lived experiences and narratives of a male African American engineer, along with pragmatic curriculum design methods, were used to reframe already vetted science, technology, engineering, and mathematics (STEM) content on the physics of roller coasters created by the National Aeronautics and Space Administration (NASA, 2003).

Using these narratives can be likened to adding literature and the arts and humanities to STEM content. According to Brown et al. (2025), literature books which promote the humanity, joy, and play of Black boys, provided opportunities for learning, making connections, and positive identity development while providing pedagogical opportunities – which they indicated was often missed when attempts at inclusion were superficial. In this study, the first author pragmatically designed the lessons using best practices in science education. These included, but were not limited to, grounding inquiry in anchoring phenomena (Alliance for Science Educators Networked Improvement Community (ASE-NIC), 2022) which were integrative and provided context to facilitate other ways of thinking (Parsons, 2019), and the use of an argumentation curricula framework to engage and capture dialogues (Osborne et al., 2004) and to facilitate content supported by the three dimensions of the Next Generation Science Standards (NGSS)– Disciplinary Core Ideas, Crosscutting Concepts, and Scientific and Engineering Practices (NGSS Lead States, 2013). The prior knowledge, lived experiences, and narratives of an African American male engineer informed which STEM content would support meaningful inclusion. The curriculum design and implementation sought to capture the essence of inclusion as defined by King and Swartz: “Inclusion—This principle refers to including all cultures and groups as the subjects of their own accounts, not as objects of others’ accounts about them” (King and Swartz, 2016, p.16). King and Swartz (2016) noted that “inclusion asserts that, when all cultures and groups are understood as substantive participants in human development, their presence is necessary, not expedient or token” (p. 16). This kind of gate breaking appears in Quinlan’s (2024a) use of pragmatic methodologies, which led to the conclusion that Black representation in science curricula has important implications for the economic pipeline, social schemas, and mental health. Thus, the role of Black people as the authors and beneficiaries of their own curricular goals was considered essential to creating, learning, and belonging in the science curricula in meaningful ways.

### Positionality of the researchers

Researcher collaborators were invited after implementation. The collaboration in this research was exploratory, including researchers to provide insights into implementation data from multiple perspectives, disciplines, and frameworks dependent on their interests, expertise, and commitments. Thus, in keeping with pragmatism, this study was a true inquiry that considered the “real-world constraints of limited time and resources” (Patton, 2015, p. 153). The researchers’ motivated thinking was central to the inquiry, as motivated thinking influences strategies used, the goal for eager judgments or clarity, interests, research questions, and evidence evaluation (Molden and Higgins, 2005; Quinlan and Farina, 2025). Rather than force a consensus that provided one unified perspective, the authors agreed that providing multiple perspectives, which also served to delineate which methodology led to specific outcomes, would prove useful and practical and in keeping with the realities, strengths, and motivated thinking of the researchers.

#### Science curricula design and development

More specifically, the first author is a Black female science education researcher, with a focused career on developing curricula content and expertise using pragmatic methodologies to situate Black heritage, narratives, and lived experiences (2021, 2024a, b), and Black scientists (Workman and Quinlan, 2023) in the science curricula. Thus, Quinlan focuses on *what works*, *how it works,* and *why it works* for curricula design, development, and implementation as an iterative process. In keeping with Dewey’s pragmatist perspective, errors become opportunities for improvement. Recognizing that science curricula and content ensure the production and promotion of White Western cultural knowledge and social capital (Bourdieu, 1977; Painter, 2010), with diminishing returns for learning that positively influences Black children’s identity formation, Quinlan embraces the inquiry and creative process, and the time it would take to create content to cater to our Black children. This is her first study inviting collaborators to provide their own perspectives and a wider range of thinking during this promotion focus—to expand the nature of vetting for the curricula, as researchers investigated what was already implemented. The addition of research collaborators was especially important in providing other theoretical perspectives, as Quinlan uses and continues to develop expertise using applied cognitive theories which support understandings about schemas as a theory of remembering, to explore the impact of knowledge and culture on student learning (Quinlan, 2012, 2019, 2026 in press).

#### Using funds of knowledge from a white female perspective

Rollins comes to this research collaboration as a White woman who grew up speaking a Southern Appalachian English dialect in a predominantly White, working-class NC community. Her family experienced health concerns, loss, alcohol abuse, and trauma related to generational economic hardship. Adults and older siblings taught children innovative “makeshifting” skills, illustrative storytelling, collectivist caregiving and housekeeping, and sustainable farming. Living below the poverty line, she did not experience formal early childhood education. However, she had invaluable educational experiences at home, received a public-school education from kindergarten through twelfth grade, and attended a public state university as a first-generation college student with significant help from FAFSA. While public schooling effectively prepared her for careers in social work and early childhood education, her family’s language and literacy practices were viewed as inferior or “wrong” and essentially “schooled out” of them beginning in kindergarten. During graduate studies at a private university in NYC, Moll et al.’s (1992) Funds of Knowledge (FoK) framework helped her further analyze the impact of her families’ language and literacy skills and overall resourcefulness as an early childhood educator.

Like all other research collaborators except the first author, Rollins was not involved in determining the nature of the content or implementation but was invited because of her commitment to centering the voices of her Black students in healthy ways and with healthy boundaries, as an instructor and advisor. Rollins was especially interested in the students’ dialogues and what she observed in the videos, which influenced the research direction and questions explored. Both her methodology and predisposition considered that over 80% of teachers are White, and the field of science education also reflects these widening demographic disparities (NCES, 2015–2016, 2018).

#### Critical perspectives in science education

Likely was the only other collaborator who attended the live sessions with the participants. As a Black woman scientist and science educator from the southern United States, Likely’s racial, cultural, and historical identities shape how she engages in science education research. Her experiences across after-school programs, K-12 classrooms, undergraduate science instruction, laboratory research, and science assessment development continue to reveal the ways Black students are marginalized in science spaces shaped by Whiteness (Sammel, 2009). These lived and professional experiences guide her commitment to creating science learning environments that honor students’ cultural capital rather than requiring assimilation (Bang et al., 2012; Yosso, 2005). Grounded in social constructivism, she understands knowledge as co-constructed and culturally mediated (Nasir, 2011). Likely views this research as part of ongoing efforts to rethink what equitable science learning can look like when it is intentionally designed *for* Black students and *by* Black people. For Likely, this project was intentionally a For Us By Us (FUBU) learning experience, drawing from Irizarry (2017) framing of education that centers the expertise, histories, and cultural practices of communities marginalized by schooling. While she shares racial and gender identities with the participants in this study, she engaged reflexively, attending to how her presence, interpretations, and commitments shape the research process. Following Probst and Berenson (2014), reflexivity is both a mindset and a set of actions that require ongoing awareness of how she influences what is being studied and how the study, in turn, shapes her. Thus, Likely brings important, critical perspectives to this study in science education research.

#### Family engagement perspectives

As a Black, African American woman, Smith (TaVshea) approaches this research through the lens of the African Diaspora, and her racial and ethnic identity profoundly influences how she understands educational systems, access to resources, and community roles in child development. Her lived experiences as a member of a historically marginalized community inform both her research interests and methodological approaches. Her K-12 public school education shaped her understanding of education as a communal responsibility rather than an isolated institutional endeavor. Smith, T., is driven by the understanding that when parents are empowered with resources, knowledge, and confidence, they become powerful advocates and educators for their children.

#### Disciplinary literacy perspectives

Smith (Delphia) approaches this research as an Afro-Caribbean immigrant woman of color raised in a blue-collar, working-class community in what is considered an urban community. As a first-generation college student, her educational journey included both public and private schools and undergraduate study in her home country, followed by graduate school in the United States. These experiences helped to shape the educator she is today. Her disciplinary training in literacy and mixed research has influenced her research focus. She believes that the U.S. is currently in a literacy crisis. Her research centers on this area and seeks to improve reading outcomes of U.S. children. In this study, she draws on her expertise in literacy, specifically disciplinary literacy.

### Goal of the research

Therefore, this research sought to understand the benefits from multiple perspectives, disciplines, and frameworks by creating a community of researchers who explored and examined the dialogue data and videos derived from the design and implementation by the first author. Researchers’ expertise and experience using specific frameworks to explore and explain data, as well as the research interests and questions for which sufficient data existed, influenced the direction of the study. Given the time constraints, this research was pragmatic in using the cultural and knowledge resources of the collaborators who engaged in continuous discussions leading to the following research questions:

(1) What knowledge patterns and connections are observed using schema theory and supporting frameworks to explore dialogues during implementation?
(2) In what ways did the Roller Coaster Physics curriculum invite Black elementary and middle school students to showcase their funds of knowledge as related to STEM?
(3) What were the benefits of including the lived experiences and narratives of Black heritage and Black people in meaningful ways in the science/STEM curriculum? What ideas and themes were salient to the researchers?

## Literature review

The literature explored was in keeping with the multiple perspectives and critical and conceptual understandings of the researchers as it related to the implementation. Creswell (2007) noted that “individuals holding this worldview [a pragmatic worldview] focus on the outcomes of the research—the actions, situations, and consequences of inquiry—rather than antecedent conditions (as in post positivism)” (p. 22). The literature explored therefore reflects critical understandings of why this matters, the importance of cultural knowledge, connections to Black homeschooling and family engagement, schema theory as a conceptual framework, and lastly, Funds of Kowledge as a conceptual framework.

### Cultural and heritage knowledge in the curricula

This research sought to address what Bourdieu and Wacquant (1992) describe as cultural arbitrariness, but this case, in the science curricula. Bourdieu and Wacquant (1992) used the term “cultural arbitrary” to describe how intellectuals might exact power by ascribing an arbitrariness or a matter-of-fact or objectiveness to ascribe specific characteristics which masks a purpose that leads to different kinds of symbolic violence.

By inserting Black representation using a similar cultural arbitrariness, this study addresses the symbolic violence exacted upon Black people through the absence of positive and powerful representations, or the use of what Mutegi (2013) describes as negative psychological antecedents. The inclusion and creation of multimedia products that captured the lived experiences and narratives of an African American roller coaster engineer served as an entry point for learning about the science, technology, and engineering, math, and non-science connections. This served as an entry point by eliciting students’ prior knowledge, generating curiosity and questions, and shedding light on students’ concepts rather than simply eliciting “right” answers or one solution (Bybee et al., 2006). Embedded within these narratives were not only the phenomenon of roller coasters or theme parks, but also the engineer’s experiences and memories as an African American male. King and Swartz (2016) noted that “heritage knowledge refers to group memory, a repository or heritable legacy that makes a feeling of belonging to one’s people possible (Clarke, 1994; King, 2006)” (cited in King and Swartz, 2016, p. 4) and “cultural knowledge is knowledge gained about the cultural legacies and patterns in cultures other than one’s own” (King and Swartz, 2016, p. 4). In this study, culture is loosely defined by the shared experiences, shared memory, and shared heritage that characterize the lived experiences of Black people in a racialized world, leading science education researcher Walls (2016) to conclude that the experiences of Black people are unique enough to warrant separate study. This study drew on this cultural knowledge, “group memory,” and, therefore, the Black male engineer’s lived experiences as an entry into learning science, engineering, math, and other STEM concepts, with connections to literacy. Moreover, this work addresses what Ladson-Billings et al. (2024) noted was missing from curricula that purport to be culturally relevant or culturally sustaining: a focus on the people who make up the culture and allowing the people to define what is culturally relevant or sustaining.

Enciso and Ryan (2011) noted that for all students “the potential to learn is optimal within situations where a problem makes use of and extends the language, knowledge, motivation and relationships already available to learners” (p. 133). What this means for Black student learners is that curriculum development needs to consider the relationships that already exist for Black student learners and to recognize that their prior knowledge and motivations are already available to them. One problematic area is the lack of entry points into learning science, which affects student learning and sense making. Put another way, learning opportunities require building appropriate schemas.

A learning ecosystem was specifically created to support learning and belonging by capturing science content knowledge, heritage knowledge, and cultural knowledge. King and Swartz (2016) noted that “by using content and pedagogy that draws upon heritage knowledge-and the worldviews students’ heritages reflect we can provide comprehensive instruction and locate students culturally” (p. 5). This study therefore provided an example that teachers could model with children, while highlighting the positive attributes and cognition of the Black community and of Black children, which Ladson-Billings (2021) noted were missing from the field, making it challenging for teachers to know what to do.

### Black homeschooling community and family engagement

Thus, this research brings the community engineer into formal lessons and formal lessons into the community space for homeschoolers to define what is relevant. Compared to formal educational settings, informal learning contexts can serve as counterspaces that more fully meet the needs of Black students. Homeschooling provided a unique space to build a learning ecosystem that engages parents and the community, one of the six strategies the Committee on STEM Learning is Everywhere (National Research Council, 2014) noted was important. They found that none of the 15 STEM learning ecosystems they looked at, including the afterschool programs, engaged all six of the strategies they found important: 1. equipping educators, 2. bringing educators from different sectors, 3. linking in-school and out-of-school learning, 4. learning progressions, 5. inquiry, project-based, and real-world connections, and lastly 6. engaging families and communities (p. 18). The Committee diagrammed the concentric circles of influences that surround the learner; the inner circle closest to the learner shows caregivers, role models, friends, family, peers, and mentors. Then the next outer circle included the internet, media, hobby club, library, church, sport, Boys and Girls Club, 4-H, After-school, CBO, school, and museum. The outermost circle was regulations, culture, social cohesion, community, values, norms, funding, policies (National Research Council, 2014, p. 5). The homeschooling ecosystem in the present study, unlike all other ecosystems, provided a unique opportunity to engage both learners and those in their most immediate circle of influence, who could themselves become a part of the learning ecosystem.

While this work did not fully capture the relationships available to students, family engagement was both central and peripheral to this work. Without family engagement, this work would never have happened. Therefore, this research forecasts the critical need to understand how families, particularly African American families, serve as essential contexts for science learning and how educational institutions can better support their engagement in STEM education. Family engagement in education represents a complex, multifaceted construct that extends far beyond traditional notions of parental involvement. The theoretical foundation for understanding family engagement draws heavily from sociocognitive and sociocultural perspectives on learning (Gonzalez et al., 2005; Gutierrez and Rogoff, 2003; Hutchins, 1995; Lave and Wenger, 1991). These frameworks position knowledge and knowing as inherently social, cultural, historical, and situated within specific contexts, constructed through meaningful interactions and experiences. This positioning allows us to think differently, understand the strengths of these communities, and examine how the lived experiences and narratives of Black people enhance the curricula and what is missing or deficit in the existing science curricula.

### Funds of knowledge as a conceptual framework

Building upon the work of Vélez-Ibáñez and Greenberg (1992) and constructing a framework for leveraging children’s Funds of Knowledge (FoK) in school settings, Moll et al. (1992) sought to “portray the complex functions of households within their sociohistorical contexts” (p. 132). Although their ethnographic studies began in the Southwestern region of the United States among Latino populations, Moll et al. (1992) have encouraged educators and education researchers over the decades to build on the FoK framework and methodology (Llopart and Esteban-Guitart, 2018)—with the aim of forging communities of learners.

Among the numerous studies taken up within and outside the U. S., the FoK framework has been utilized in teaching to “enhanc[e] the curriculum with multicultural perspectives and involv[e] parents in the educational process” (Olmedo, 1997, p. 45), to connect content to what learners have already experienced and know (Sleeter, 2005), to “analyse and understand young children’s play interests through a sociocultural lens” (Chesworth, 2016, p. 295), to “integrate attention to children’s thinking” (Turner and Drake, 2016, p. 41), and to determine the ways that “learning environments serve students, particularly those who have been historically marginalized or ignored” (Denton and Borrego, 2021, p. 71). While key studies are categorized below, their richness allows them to encompass a range of theory-informed teaching practices.

### Enhance the curriculum with family involvement

Countless researchers have written about the use of FoK, particularly focusing on family involvement, to create both more authentic and more accessible curricula. For instance, soon after Moll et al. (1992) seminal study was published, Olmedo (1997) studied the significance of sharing and leveraging family funds of knowledge through oral history. Specifically, this researcher used a case study and relied on interviews to explore how the curriculum can include family members’ lived experiences to promote students’ learning about our multicultural society. Aiming to analyze how Funds of Knowledge were included in both school and out-of-school mathematics, Civil (2016) also engaged in an exploratory study using occupational interviews to address the questions, “How do we define competence? How do task and environment affect engagement? What is the role of affect, language, and cognition in different settings?” (p. 41). Through her study, Civil drew upon developing theories in connection with her own research as she discussed the practices and processes that can be evidenced through a Funds of Knowledge approach to mathematics. A significant finding from a family participant highlights how family members want to identify themselves as teachers who have a voice in their child’s learning. Olmedo (1997) and Civil (2016) both contextualize their research within a landscape where the experiences and knowledges of Communities of Color have been marginalized. While goals for oral history and occupational interviews have continued to shift, these authors note that a recurring objective is to ensure all students can meaningfully connect to the content by leveraging their family funds of knowledge. This connection is also fostered by connecting curriculum content to learners’ existing interests and expertise to build schema.

### Connect content to existing interests and expertise to build schema

In her interpretive study in an early childhood education context, Chesworth (2016) explored how educators can leverage children’s use of FoK to build collective schema. In this study, “the interpretations of play offered by the children and parents illuminated the diverse ways in which children’s sociocultural experiences underpinned their play interests” (Chesworth, 2016, p. 305). Findings support “possibilities for children to co-construct complex and creative play” (p. 306). Although this study focused on young children, it has implications for playful learning in STEM subject areas through elementary school and beyond. These understanding led to the research question: *What knowledge patterns and connections are observed using schema theory and supporting frameworks to explore dialogues during implementation?*

### Create learning environments that serve diverse students

Volman and Gilde’s (2021) mixed-methods quasi-experimental study with elementary-aged students and their teachers explored how teachers in an intervention group enacted plans to intentionally recognize and include children’s FoK after learning about the theory, research, and related teaching practices. Through logbooks, interviews, and focus groups, researchers found that “teachers and students reported effects on students’ engagement, learning behavior, learning attitudes, collaboration skills, self-confidence, general well-being in the classroom, ambitions and perspective taking” (p. 1) along with an overall enhanced learning climate.

Through her research in partnership with Black and Latinx communities, Thevenot (2021) contends that a culturally responsive “curriculum design recognizes the varied cultures of students in the classroom and is grounded in culturally responsive teaching, with the added layer of modifying teaching strategies in the STEM classroom” (p. 61). In this participatory action research study, Thevonot co-designed and implemented a curriculum alongside co-teachers to construct a more inclusive and equitable curriculum for an afterschool PreK-12 STEM program serving diverse children. Findings from forum observations and interviews highlighted how diverse students demonstrated stronger STEM identities; a sense of achievement with STEM tasks; and overall positive relationships with teachers, co-learners, and content through their engagement in this co-designed curriculum. These studies point to a need for qualitative analysis when seeking to authentically recognize and include children’s FoK in STEM learning experiences such as roller coaster physics. This literature led us to the research question *In what ways did the Roller coaster Physics curriculum invite Black elementary and middle school students to showcase their funds of knowledge as related to STEM?*

### Schema theory as a conceptual framework

Schema theory and an understanding of schemas provided an opportunity to capture both concurrent and retrospective thinking, which captures what is remembered, what is learned, and how the information is used (Marshall, 1995). Schemas refer to organic reactions and the active organization of knowledge and experiences that depend on past experiences (Bartlett, 1932), and this study captured them in a learning ecosystem that integrated formal and informal learning and researchers’ institutions with homeschooling. This research sought to make learning accessible by increasing the likelihood that students could connect what was learned with the motivation and relationships already available to them—their families and members of the Black homeschooling community. Piaget and Inhelder (1969) noted that when children encounter information, they may assimilate it if it is accessible or accommodate it if it is not congruent with their pre-existing knowledge and relationships. Put another way, Sakamoto and Love (2004) noted that schemas function as filtering devices determining what is assimilated or accommodated. This may or may not lead to distortion, as Kahneman and Frederick (2005) noted, because people answer a version of a question shaped by their interpretations or prior schemes. Research shows that for Black children, the opportunity to learn is less available to them and that they are more likely to learn in quiet isolation than in an interactive and more social setting than White children (Boykin and Noguera, 2011). Piaget and Inhelder (1969) noted that:

The decentering of cognitive constructions necessary for the development of the operations is inseparable from the decentering of affective and social constructions. But the term ‘social’ must not be thought of in the narrow sense of educational, cultural, or moral transmission alone: rather, it covers an interpersonal process of socialization which is at once cognitive, affective, and moral (Piaget and Inhelder, 1969, p. 95).

This study brought formal lessons into a learning ecosystem to capture the strengths and advantages of both the curriculum content and learning in this ecosystem. However, in seeking to understand both students’ funds of knowledge and their schemas, this study sought to capture both the collective and individualistic. Bandura (1997) cautions against focusing only on the collective as “these global classifications mask much diversity and variability” (p. 31), even as cultures “rest on a questionable uniformity assumption” (p. 31). Therefore, this study considered not only the schemas of the participants but also the shared goals of the researchers (see Appendix B). Molden and Higgins (2005) noted that people evaluated evidence that supported positive attributions about themselves, attended to questions, and used research strategies similarly, as researchers may be motivated by goals such as promoting inclusion or preventing omissions (Molden and Higgins, 2005; Quinlan and Farina, 2025).

While research on schemas has been debated, this study used Marshall’s schemas, which were initially operationalized for Mathematics but have been used repeatedly by the author, Quinlan (2012, 2019, 2024a) in science education research. Marshall’s (1995) work on schemas operationalized four knowledge types active in student learning: identification knowledge, elaboration knowledge, planning knowledge, and execution knowledge. According to Marshall, identification knowledge begins when students recognize or make connection(s) with an idea and is usually accompanied by pattern recognition. Depending on the connection students make, they may develop elaboration knowledge by hypothesizing about a situation or abstracting from experiences. Planning knowledge reflects the plans students create using that information, which may lead to execution knowledge attached to a useful skill, depending on the goals for execution.

Put another way, learning opportunities require building appropriate schemas. Schemas take on several forms and can be characterized by “architectural features,” such as *storage*, *networks*, *connectivity*, *flexibility*, *variability in size*, and *embedding* (Marshall, 1995). Marshall noted that more connections between schemas lead to more cohesive learning. Fewer connections meant students’ learning was expected to be less cohesive, with weaker schemas for any elements within their learning. Marshall also described schemas as having “substance” or “psychological features” indicated by *schema construction*, *attention*, *repetitions*, *uniqueness*, *abstractions*, *content variability*, *neither concept nor rule*, and so on. These features could be captured in students’ organic reactions and in their dialogues to the extent that the content facilitated schema building.

The idea of building schemas is seen when we consider not only the culture, dialogues, and multiple interactions during learning, but also individual schemas already available to students along with their individual prior experiences. Bartlett (1932) noted that “whenever there is any order or regularity of behavior, a particular response is possible only because it is related to other similar responses which have been serially organized, yet which operate, not simply as individual members coming one after another, but as a unitary mass” (p. 201).

Similarly, Quinlan (2012, 2024a) found that students with prior schemas more readily abstracted from their experiences, whereas those with no prior experiences exhibited learning as keyword searchers or forceful features, rather than abstracting from or connecting with specific information. This literature led us to the research questions, which sought to understand what ideas and themes were salient to the researchers using two frameworks—schema theory and Funds of Knowledge—and what the benefits were of including the lived experiences and narratives of Black heritage and Black people in meaningful ways in the science/STEM curriculum.

## Methodology

### Research design

#### Overview and rationale

The research design does not rely on a single distinct approach to qualitative inquiry. The design is emergent, taking on characteristics of specific approaches dependent on the stage. During treatment design, the first author and researcher took on the role, thinking, and predisposition of a curriculum designer, placing herself in the shoes of both teacher and student. The introspective and interpretive nature of the content curriculum creation shared elements of autobiography, which:

Focuses on how individuals’ culture, gender, history, and experiences shape all aspects of the [curricula content development], from their choice of a question to address, to how they collect data, to how they interpret the situation, and to what they expect to obtain from conducting the research” (Creswell and Poth, 2018, p. 49).

The words “qualitative project” were replaced with “curricula content development” because, while the treatment was a part of the process, curriculum design, in some ways, was bracketed from the research design process itself. It was impossible to think like a researcher during this stage and to separate oneself from the curricula. Quinlan was simply the Black female whose heritage and lived experiences were underrepresented and marginalized in the science curricula, placing herself as learner, teacher, and design expert, creating for Black children a curricular experience which would serve them well. These thought processes also shared elements of autoethnography with “multiple layers of consciousness, the vulnerable self, the coherent self…the subversion of dominant discourses” (Creswell and Poth, 2018, p. 70). What they need is what she needed but did not receive from the curricula.

Similarly, implementation did not fit precisely into any specific qualitative inquiry. These lessons were created in the likeness of formal classroom presentations. Like action research, the lesson progression was heavily influenced by participants’ perspectives (Ravitch and Mittenfelner Carl, 2021). However, unlike action research, there was no formal data analysis in real time even as the focus was on the experiences of the students. Their experiences were used to inform and modify subsequent meetings. Their experiences were captured in the video clips and data analyses, which sought to explore the essence of these experiences in keeping with phenomenology (Creswell and Creswell, 2018). During implementation the research process became ethnographic as researchers were present as instructor (Quinlan) or participant observer (Likely), who “shared patterns of behaviors, language, and actions of an intact cultural group in a natural setting over a prolonged period of time” (Creswell and Creswell, 2018, p. 13) in this case 8 weeks.

#### Pragmatism

Creswell and Creswell (2018) draw from Cherryholmes’s notes in defining pragmatism: “Instead of focusing on methods, researchers emphasize the research program and use all approaches available to understand it” (p. 249). The use of pragmatism as a research design is in good company with those known to use it to reach their goals. According to Cherryholmes (1992), philosophers such as John Dewey, George Herbert Mead, William James, and others were pragmatists, and the hallmark of pragmatism is supported by what Dewey wrote (cited by Cherryholmes):

Pragmatism does not insist upon antecedent phenomena but upon consequent phenomena; not upon the precedents but upon the possibilities of action. And this change in point of view is almost revolutionary in its consequences. [W]hen we take the point of view of pragmatism; we see that general ideas have a very different role to play than that of reporting and registering past experiences. They are the basis for organizing future observations and experiences (1931, pp. 32–33). (cited in Cherryholmes, 1992, p. 13).

In keeping with pragmatism, this work focuses on what was considered integral to pragmatism: “Values, aesthetics, politics, and social and normative preferences are integral to pragmatic research, its interpretation and utilization” (Cherryholmes, 1992, p. 13). This was done by first ensuring that research was systematic and research methods were sound, meaning curricula were designed and implemented as the treatment, though not the focus of the analyses. This raised the following questions: What literature should we explore? What questions should we ask? What frameworks should we use? And how should we report the findings to highlight and promote best practices and benefits, along with shortcomings if they exist, though this was not the main goal? Ravitch and Mittenfelner Carl (2021) noted that building conceptual frameworks is an iterative, reflective process that requires continuously asking questions and knowing how the different parts connect with the research questions. Providing insights to the readers about what the possibilities were and what literature could forecast the future direction of this study, even as it provided some insights to inform the reader about the research and its intentions, was considered both important and pragmatic. In considering data analysis methods, one pragmatic approach was to quantify the codes and data to reflect perceived value and importance based on the accumulation of evidence over a certain time, knowing that given more time or use of a different framework might yield different results. As such, this research study is in keeping with how researchers define pragmatism.

#### Treatment

Curricula products were created by the first author and implemented as the treatment for this study. First, Quinlan hosted a podcast interview with the engineer Mr. Grady (pseudonym) to generate narratives and used the narratives to produce three themed video clips with B-roll images and videos for enhancement titled *Engineering the thrill: Designing roller coaster* (5:39 min), *Roller coaster project* (3:46 min), and *Navigating challenges: representation in engineering* (9:24 min). The video clips were used as an engaging context and phenomena to encourage student interest and questions.

Quinlan created a sequence of physics topics, lessons, and questions that supported the roller coaster narratives and understandings of related physics concepts. The topic each week provided a conceptual introduction to physics concepts in as tangible ways as possible with the goal of reaching both younger and older children; the goal was a cursory introduction to advanced ideas that older children would grasp without leaving out the younger children. Quinlan’s goal was to encourage talk about what they were understanding, learning, or connected with. Some minor adjustments were made mainly to the first and last weeks, for example to provide students with opportunity to “show-and-tell” what they built, while reviewing, applying, and making sense of all the physics terms they learned in the last week. Table 1 provides an overview of the topics and questions that were the focus for each week’s lessons. Appendix A provides a table showing how the lessons are supported by the Next Generation Science Standards and the Common Core for Math and English Language used in formal learning.

**Table 1:** Overview of weekly virtual lessons: roller coaster physics and stem connections.

Week 1	Week 2	Week 3	Week 4
Introduction to roller coasters and roller coaster engineers	Forces and Motion	Designing a roller coaster	Mathematics connections
What is a roller coaster? Who designs and works on roller coasters? Background information and history of roller coasters.	How does an understanding of forces help you to design roller coasters? How does an understanding of forces help you to understand how to design roller coasters?	How can you use your own creativity and interests, engineering design, physics, math, and other concepts to design and build your own roller coaster? How can building your own roller coaster help you to understand the STEM behind roller coasters?	How do you measure motion? What are different ways to measure motion? Determining position versus distance traveled. How do you determine and calculate speed?
Week 5	Week 6	Week 7	Week 8
Velocity and acceleration	Gravity and motion	Energy of motion	Microgravity and physics of roller coaster
Calculating and distinguishing between displacement, velocity, and acceleration	What is gravity? How does gravity affect motion?	What is energy? How does energy affect motion? What are sources of motion/different types of energy? Exceptions.	What is microgravity? Conditions for microgravity. Describe and discuss the physics of roller coasters/your roller coaster.

#### Participants

Participants were recruited from two Black homeschooling Facebook groups in the Mid-Atlantic region, in which Quinlan, a former homeschooler, was a member. No one was excluded from attending, and therefore participants also reflected a convenience sampling in their natural virtual homeschooling environment. The number of attendees ranged from 22 participants to 48 participants with an average of 35 attendees. These numbers included 2 science education researchers (Quinlan and Likely), 1 teacher guest, homeschooling student participants with ages ranging from 3 years old to 14 years old, and their parents or guardians. For the duration of the 8 weeks (with one week break in between), there appeared to have been a total of about 38 homeschooling students whose ages ranged from 3 years old to 14 years old. No attendance was taken. From zoom’s attendance record it appears that of 31 children (homeschooling students), 10 homeschooling children attended 6 to 8 times, 10 attended 3 to 5 times, and 11 homeschoolers attended once or twice. A few found out and joined the class later by word of mouth or attended once. It is possible that a few only joined the two sessions with the engineer but this was not explored.

The 6 parents who filled out the demographics form sent out after the 8 weeks identified as either Black, African American, Black American, or American Black. Length of time homeschooling ranged from 1st year (1), 2 years (2), 4 years (2), and 3 to 4 years (2). Reasons for homeschooling varied from the local school district not meeting expectations, to “quality time with my children. I wanted to build confidence and independent thinking,” continuing homeschooling after Covid “so that there would be no limitations on their learning,” to being a Christian and feeling led by the Holy Spirit to do so leading this mother to quit her four jobs to stay home with her son while her husband continued his corporate job. Table 2 provides a data summary of focal participants reflected in the findings. The table provides pseudonyms which reflect traditional male/female gender roles. One limitation of this study was that participants were asked how they identified by Race and ethnicity but were not asked how they identified by gender. Ages were derived from labels they provided to help with grouping activities by age. Phenotypically all children were Black or African American in appearance. It is important to note that parents were also in attendance. Parents signed consent forms, with institutional IRB approval prior to being sent the zoom link. Parents were either in attendance in the same screen but in the background, or some parents joined separately with videos off. Parents were reminded of the risk to privacy and encouraged to use only their children’s first name or a pseudonym or to keep cameras off for privacy concerns. Parents were reminded that the videos would be shared, used in research, for training teachers, and to promote the interests of Black people and Black children.

**Table 2:** Focal participant data—participants either identified by or were identified phenotypically as black/African American heritage.

Pseudonym	Age/grade	Pseudonym	Age/grade
Zina	7 yrs	Wonder ride	9 yrs.??
Maurice	13 yrs	Musa	12 yrs
Ebony	8 yrs. old??	Deshawn	10 yrs
Janiyah	11 yrs. 5 mths	Ella	11 yrs./6th grade
Xander	9 yrs	Amani	13/7th grade
Mariah	10/6^th^ grade	Serenity	14 yrs
Lola	6 yrs. old	Kam	9 yrs./4^th^ grade
Halle	5/Kindergarten	Zana	9 yrs.??
Sarah	14 yrs	Dr. Catherine Quinlan	Instructor
Malika	6 yrs./2^nd^ grade	Ms. Williams	Guest Teacher
Marquis	8 yrs	Mr. Grady,	Engineer

“??” not reported/guessed | not all grades were reported.

### Data collection

Data collection reflected students’ organic and active contributions and reactions in the recordings with no prior preparation or rehearsal. It is important to note that no prior preparation or training was provided to the educators who took part in the curricula implementation. The study relied on their predispositions and respect for Black children, eagerness and desire to participate and contribute, their content knowledge and education expertise, and responsiveness to the students.

Eight 1-h Zoom video recordings were the main source of data collection. The Zoom recordings generated an enormous number of line-by-line transcripts totaling 955 pages with 99,435 words. Most of the researchers used this vast amount during their analyses. After using this partially it was condensed to 181 pages with 65,234 words before the final set of analyses. Additional video recordings were generated from the breakout rooms and focus groups during the last day which were transcribed separately but were not included in the final analyses. Additional videos were used only for triangulation. Both the video recordings were used in conjunction with the transcripts to correct for transcript errors and to make observations not derived from only reading the transcripts. Attendance information was derived from the Zoom attendance reports generated. One drawback was that the initial Zoom settings did not allow for group observations of the entire class but only showed who spoke.

### Data analysis

First, each researcher used preliminary coding or jottings, or pre-coding (Saldaña, 2021), prior to looking at the literature to determine questions to explore and the best frameworks and literature that explore and explain the data from different lenses. In the beginning, the researchers met almost biweekly on Zoom to discuss the next steps for analysis and framing. Then the schema theory, along with the anchor competencies, was used to explore what knowledge patterns and connections were observed in the dialogues during implementation. Funds of Knowledge was used as a framework to explore what ways the Roller Coaster Physics curriculum invited Black elementary and middle school students to showcase their funds of knowledge as it related to STEM. Finally, the third question on the benefits was explored by making connections between the findings using the different frameworks.

### Analysis using Schema theory

During analytic coding, the role of the instructor in driving these goals became imperative. It became difficult to separate the nature of pattern finding demonstrated in student learning from the initial goal to elicit a connection with the content and context. Therefore, codes from the emotional, cultural anchor competencies, which were designed for teacher moves, were increasingly explored and included. In seeking to understand students’ experiences and connections, it became apparent that students’ responses went beyond cognitive learning and reflected their affective learning, reflective of how they received, valued, or interacted with the information. Therefore, Marshall’s knowledge schemas (see Table 3) were combined with Markowitz and Bouffard’s (2020) Social, Emotional, and Cultural Anchor Competencies. Since all but one category (“Responding constructively across differences category”) was used in the analysis, permission was sought to include the diagram in this study (see Figure 1). The saliency of the categories in the framework was used to describe the nature of identification, elaboration, planning, and execution knowledge, and as separate from these schemas. The categories in the framework were interpreted using the science education and applied cognition lens of the first author and without Markowitz’ accompanying descriptions, which were explored after analysis.

**Table 3:** Replicated table of guiding questions from Marshall’s (1995) schema depiction and knowledge types (Quinlan, 2019).

Identification knowledge (IDK)	Elaboration knowledge (ELK)	Planning knowledge (PLK)	Execution knowledge (EXK)
What type of IDK do students exhibit? What patterns do students recognize? What schemas are activated? What is the evidence that this particular schema is activated? What did students have to identify in the task? What is the situation, event, or experience? What are the main features of the situation or event?	What declarations were made? What specific examples of individuals’ experiences were made? What general abstractions were described from these experiences? What verbal and visual information is retained? (What is the evidence of retention?) What details from current experiences were fit into a template about the situation? What template did students create about the situation? What hypotheses were formed through application of IDK? How do students evaluate hypotheses to determine if sufficient evidence exists to warrant recognition? What new verbal and visual details were students expected to learn? How do students acquire both aspects of ELK? What situations were drawn on/heeded to be elaborated?	How can a schema be used to make plans and create expectations? How can the schema be used to set up goals and subgoals? What knowledge is acquired from experiences in using each schema? What necessary knowledge did students develop to help them examine and understand the problem? To what extent did students gain a perspective, i.e., a broad perspective of making plans that took them from the beginning to the end? What is the evidence that students have PLK and not just IDK and PLK?	What knowledge is required for students to carry out the final steps of the plan? What techniques led to actions such as performing the final skills required for the end goal/end task? To what extent did students understand when and why to carry out various plans?

**Figure 1:**
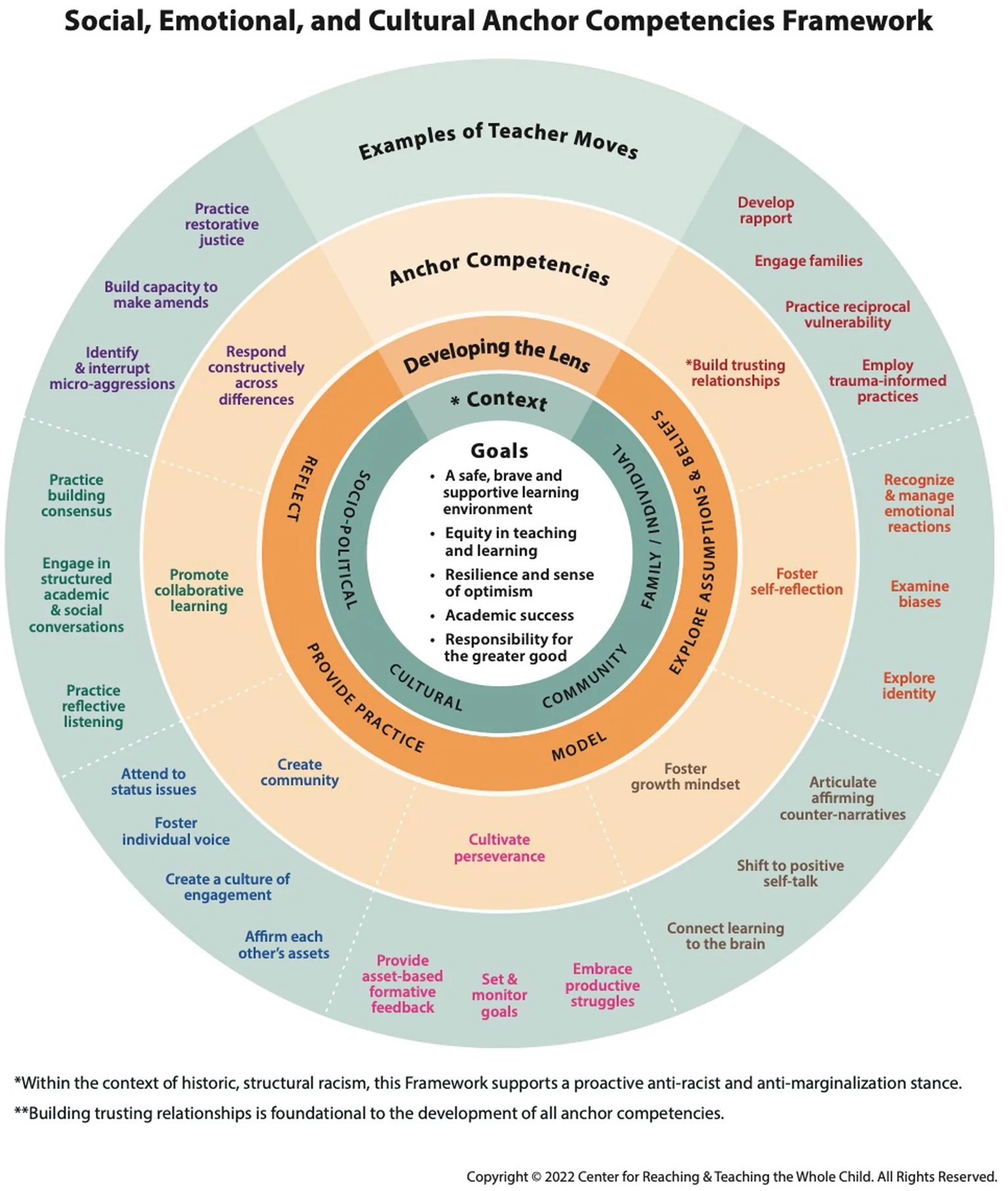
Social, emotional, cultural, anchor competencies framework. Replicated from Markowitz and Bouffard (2020) with permission from the author.

The codes were not necessarily used as they are described in support of this anchor wheel, but rather provided an analytic lens in keeping with what was observed. For example, the code *community—foster individual voice* provided descriptors for what was observed in the data.

NVivo software was used in this analysis, resulting in an enormous number of codes generated using both schema theory and anchor competencies. A total of 57 codes were generated in the initial analysis using only Zoom transcripts. The use of the codes was widely represented in one to 8 of the sources, with each week’s transcript as one source. A total of 874 references were created in the preliminary analyses, which included references that overlapped with more than one code. The first 17 codes with the highest number of references were used to begin the triangulation process. A matrix coding query was done in NVivo to determine overlap between codes. A map was created for each of the 17 codes and used to create a comparison matrix table to map out the two or three triangulation codes that would be used to explain the findings. A table was created to visualize the three most abundant overlaps, which increased to four and then five overlaps. Codes were added until recurrences were evident, resulting in 11 comparison rows with three to five overlaps. It is important to note that the numbering of the references for specific codes points to what was salient to the researcher, which varies from researcher to researcher. Numeration of codes was used to add another dimension that showed saliency and importance to the researcher and to the data. Finally, a matrix table with the overlaps was used to generate queries that explored connections between references and to build the narratives. This was done until repetition yielded nothing new from the data.

### Analysis using funds of knowledge

The range of data sources (e.g., video recordings with transcriptions enabled, line-by-line transcript documents, curriculum materials such as slides and videos, and memos documenting communication with parents/caregivers) was analyzed leveraging a Funds of Knowledge lens. Transcript documents were the primary data source, with video recordings, curriculum materials, and memos referenced for triangulation. Data analysis began with open coding; axial coding was utilized due to the range of data sources and complexity of the data.

Axial coding is a qualitative research technique that involves relating data together in order to reveal codes, categories, and subcategories grounded within participants’ voices within one’s collected data. In other words, axial coding is one way to construct linkages between data. Axial coding has a historical relationship with grounded theory, which is a qualitative methodological framework that involves constantly comparing emergent themes within one’s data set in order to make theoretical claims regarding one’s communicative conduct. Coding, or the process of inductively locating linkages between data, may occur in myriad ways such as behaviors, events, activities, strategies, states, meanings, participation, relationships, conditions, consequences, and settings, to name a few (Simmons, 2017, p. 80).

Transcript documents were coded beginning with the first 5 weeks. Beginning with open coding, a member of the research team closely viewed and listened to the first 5 weeks of videos while jotting initial impressions. Transcripts were then read line-by-line, beginning with Week 1, and words and phrases that were repeated and/or informed by initial impressions were highlighted.

During this process, highlighted colors were revised (color-coded) as patterns took shape. Codes were defined only after the full transcript for Week 1 had been carefully analyzed. Additionally, reflective notes were jotted while coding (e.g., connections to the literature on Funds of Knowledge, questions for clarification—to be answered by reviewing additional data sources, and questions for researchers who participated in the weekly sessions). The labeled codes were then categorized in a final review of the Week 1 transcript. Axial codes (e.g., *Assisting with learning (family/adults); Connecting to/between learners; Showing interest/enthusiasm; Giving directions/sharing information; Positioning self as co-learner; Setting up STEM learning objective; Aligning response with STEM learning objective; Sharing expertise aligned with STEM learning objective*) were applied to a line-by-line review of Weeks 2–8, as video recordings and transcript documents were populated in our shared folder. Referring again to initial impressions and considering components of *Funds of Knowledge* (e.g., *positioning self as learner, connecting/to between learners, sharing expertise aligned with learning objective*), the overarching themes that emerged were: *Community of Learners* and *STEM Language*. These themes will be discussed below in the findings.

### Validity and credibility

Checking and refining the codes was done continuously. It became clear that using Marshall’s knowledge types would be insufficient to explain schemas and connections observed from the actions and reactions. A modification of the categories from the social, emotional, and cultural anchor competencies added explained what was observed in the data. The codes were refined, revised, and reorganized before being grouped into common themes. This study used *“multiple coding* (Barbour, 2001), which means multiple researchers coding the same data to look for shared understanding,” (Ravitch and Mittenfelner Carl, 2021, p. 285). All researchers participated in initial coding to “remain open to exploring whatever theoretical possibilities we can discern in the data” (Charmaz, 2006, p. 47). The first and second authors led the formal coding using schema theory and Funds of Knowledge frameworks. All researchers looked for “disconfirming evidence, negative cases, discrepant data, or outliers” (Ravitch and Mittenfelner Carl, 2021, p. 285) from the codes and themes generated. The separation of the findings to match the frameworks and questions pursued was done to attend to the credibility, transferability, and dependability during the research process. Credibility “not only seeks complexity but also attends to the real-life complexities that exist,” transferability “provide(s) enough contextual data and framing for others to be able to fully contextualize the study’s findings,” and dependability allows methods and findings to be mapped to the research questions (Ravitch and Mittenfelner Carl, 2021, p. 269).

Counting within qualitative research can be used as a pragmatic approach to promote credibility among those who prefer numbers, and it also “shows the widespread nature of the phenomenon” (Patton, 2015, p657). Credibility within the findings is also promoted by including multiple researchers and multiple frameworks to show similarities and differences. This research invited collaborators who in some ways operated in a “critical friend role” (Patton, 2015) and as advocates for community engagement, expressing enjoyment and love for the community, and ensuring the interests of the participants in the community were also served.

Evaluative feedback provided by the focus groups with the children, the individual interviews with the engineer and the teacher, and co-interviewing between the two researchers who participated took place after the classes were over. The feedback supported the direction of the findings and in some ways might have also helped set the tone for the analyses and interpretations by the two researchers who participated in the implementation, even though the analyses focused on the dialogues. The evaluative feedback in some ways provides a “reflective triangulation” (Patton, 2015) for the direction and tone of the analyses for both the first and third authors who participated and/or viewed the feedback interviews and focus groups. The focus group with the children also provides “theory triangulation” (Patton, 2015) and justification for the need for social, emotional, and cultural connections. One limitation of the study was not eliciting additional feedback from the audience after the analyses were over. Some of the feedback from the teacher, engineer, and children was added after the analyses in the summary of the findings to show and prove this triangulation with participants’ explicit observations and experiences.

### Limitations and generalizability

When students were introduced to the video clips of the engineer during the first class, the Zoom settings and sound were problematic, leading to individual viewing with links shared, rather than group viewing as originally planned. Therefore, not all students may have viewed all three videos. Coding fatigue and the enormous amounts of transcripts led to a focus on salient ideas and themes, and some data and narratives will not be reported in this study. Since the goal of this research was to understand how and why rather than how often, the focus returned to doing more comprehensive analytic analysis about the significance of each code and making sure that repetitive coding supported and justified the narrative outcomes with a focus on saliency. As Miles et al. (2020) noted: “validity is a contested term among selected qualitative researchers” (p. 306), with some opting to focus on coming to “a deep understanding instead” (p. 306). Similarly, this research considered the subjective nature of research by paying attention to the influences of positionality on the outcomes (see Appendix B) and in predicting the nature of the outcomes using different methods of analyzing and reporting the data. While some consideration was made to merge the data, we concluded that reporting both narrative form and categories provided a deeper understanding and brought attention to the importance of these categories.

The different approaches led to different perspectives based on the different lenses and frameworks of the researchers and are not generalizable. This research predicted that the outcomes using both frameworks would complement each other but that the approach to sharing the outcomes was researcher-specific. Therefore, the findings reflect a narrative approach to reporting using the Funds of Knowledge framework as well as a categorical approach to reporting using codes which reflect cognitive processes. For reliability purposes and to show how both results complement each other, the findings utilize both approaches as if providing two reports rather than merging them as is commonly done. Participants’ evaluative feedback was also added for triangulation and reliability.

## Findings

The findings provide different ways of looking at and reporting the data using schema theory supported by the social, emotional, and cultural anchor competencies as frameworks, and Funds of Knowledge as a framework, then summarize the overall benefits of including the lived experiences and narratives of Black heritage. Part 1 of the findings shows that using schema theory as a framework with the social, emotional, and cultural categories highlighted the finer strokes of the data and pointed to finer benefits and categories such as fostering individual voice, reciprocal vulnerability, creating plans, and identifying connections with science content and formal learning, connections and proximity to Blackness, and engaging parents, to name a few. Part 2 of the findings shows that using Funds of Knowledge as a framework provided a broader stroke and broader perspective about benefits to the community of learners, learner-centered teaching strategies, and STEM language, which is reported in narrative form. Part 3 summarizes the overall benefits of including Black lived experiences and narratives based on a summation of the findings.

### Part 1. Knowledge, schemas, and social, emotional, cultural interactions

Using schema theory as a framework revealed knowledge patterns that involved *creating plans, identifying connections with the science content and formal learning,* and *connections* and *proximity to Black children and to Blackness,* among others. Schema theory also showed connections to themes that promoted a safe and supportive learning environment, as expressed in student dialogues such as encouraging creating plans, fostering individual voice, reciprocal vulnerability, affirming assets, and engaging families, to name a few. Figure 2 shows the distribution of the codes (themes and ideas) among the sources and references.

**Figure 2:**
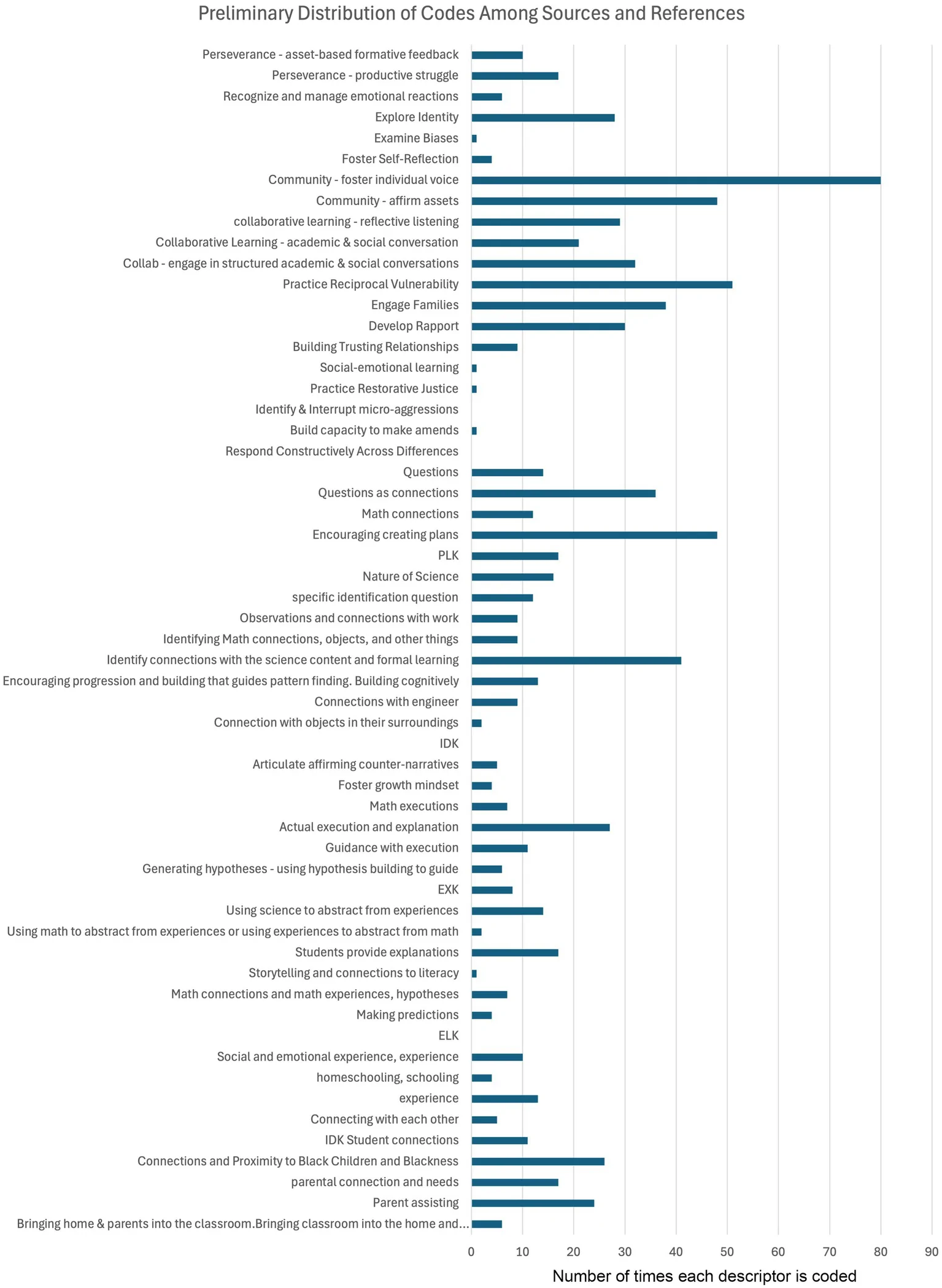
Frequency with which codes are highlighted in all dialogue data.

There was overlap between themes, with the most abundant being *foster individual voice,* which displayed the most overlap with “students provide explanations*”* and *actual execution and explanation.* Figure 3 provides a condensed diagram of overlaps observed.

**Figure 3:**
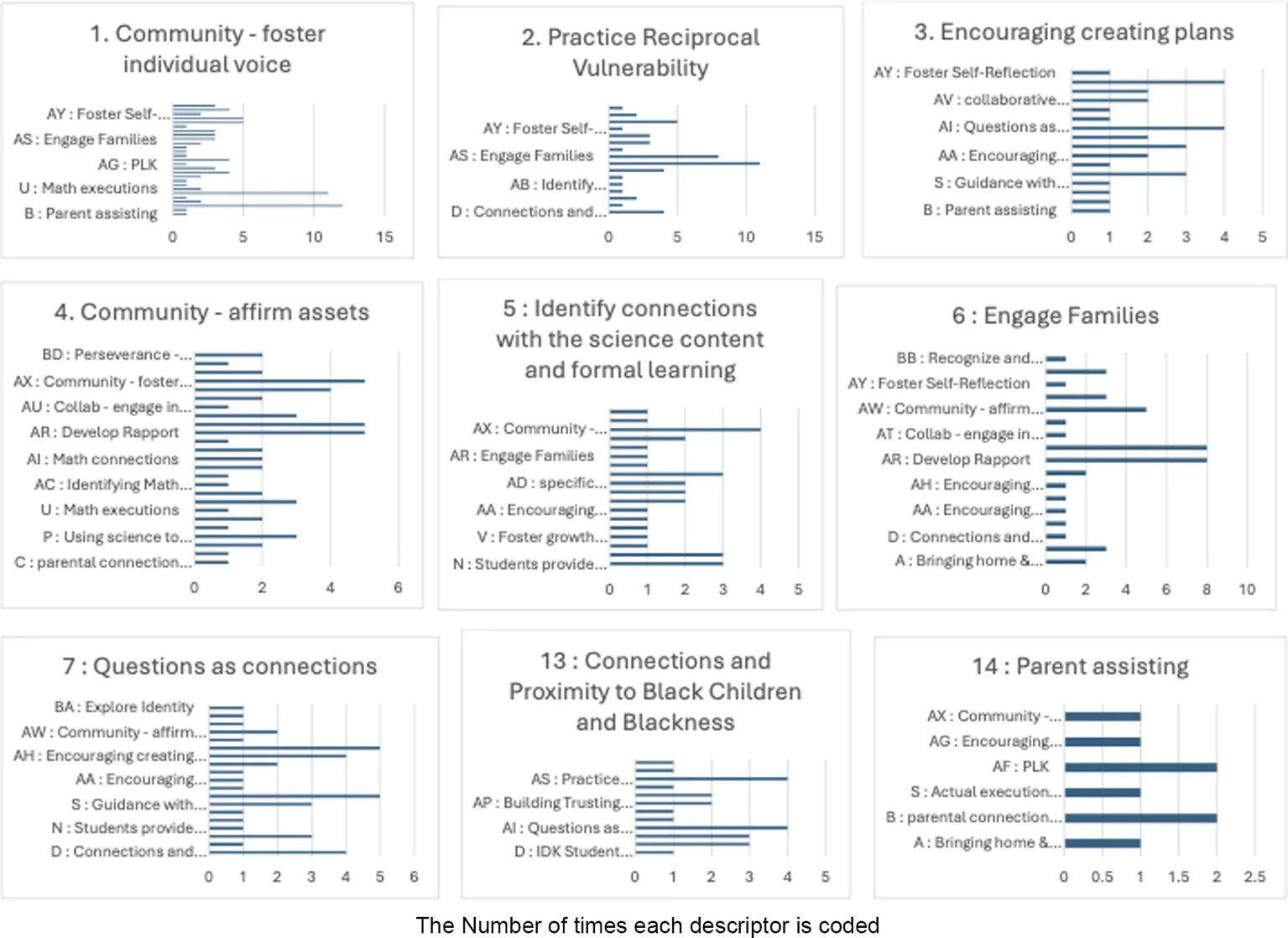
Frequency diagram showing which codes the main categories overlap with.

The second most abundant *reciprocal vulnerability* overlapped with *engage families* and *develop rapport*. *Encouraging creating plans* (48) was the third most abundant code and overlapped with *questions as connections, community-foster individual voice, identify connections with the science content*, *and formal learning*, among others. In the section that follows, the themes are used along with their overlaps, highlighted in italics, to create the narratives which follow.

### Foster individual voice

Creating community occurred through *common goals* as facilitated by the *questions*. One of these *common goals* was *fostering individual voice,* such as when the engineer asked a child to clarify her interest, “Wow, so what type of roller coaster do you have in mind that spins? Can you describe that for me, please?” He could have carried on by creating scenarios about many different types of spinning rather than homing in on her interest. Getting to know the engineer was a common goal. *Seeking community* was a goal which led to creating a new avenue in which the community guest teacher, Ms. Williams facilitated getting to know each other with questions such as, “Would you rather watch the same movie every day, or eat the same thing?” Students’ responses varied with “I should say a movie every day because I don’t like going out everywhere, even though I like to do a lot of things,” or “that’s a hard one…cause I have favorite food, but I also have favorite movie I feel like either…I feel like both.” Parents had expressed a desire for opportunities for their children to get to know each other in their homeschooling environment.

Students’ *actual execution and explanation* were central to their learning and interacting. During the predict-observe-explain activity, students’ articulation of their predictions and accounts of what happened when they blew up their balloons and released them were important because our on-screen views of them were hampered: “So, when I blew up my balloon, it went in a spiral. It went around and around, and then it went over to the door we have, and… fell on the ground right next to the door,” Zina. Mariah noted: “It went to the left corner at the top, and the air kind of propelled it more up as it went… as the air flowed down. The second time, I did it straight up, and it just went up, and then came back down after, like, a few spirals a couple times, and the third time, it just went up behind my dresser,” Mariah. Both students were specific about where it landed in their home.

Providing explanations was important for the instructor (Quinlan) to know if students were observing specific patterns as indicated by the explanation. Marquis noted, “That the air is coming out. And the balloon always goes the opposite direction, so that means the balloon is going the opposite direction.” When dropping objects such as a rubber duck, they articulated what they did, what might have gone wrong, and their timing. Malika stated, “I dropped my pencils and envelope. I felt like they… I dropped the pencils first, and then the envelope… And the pencils came down before the envelope.” Quinlan responded, “What happens if you dropped them together, at the same time?” Malika surmised, “Maybe, it would like, come down at the same time.” Guiding Malika to experiment further, the instructor asked, “Can you try that? Try that and tell me what you get, okay?”

It was also important to the instructor that students used and articulated the spatial language needed to understand what was observed through *repetitions*. This articulation occurred after they explored velocity and acceleration using simulations. They noted: “The velocity as a negative number, it does not move; it’s like it’s trying to go backward,” or “it’s changing direction when you put the negative, right?” or “if you put the acceleration on… on zero, it’s gonna not… it’s not gonna go… it’s gonna go slow, and it’s not gonna, like, move anywhere. It’s gonna move, but it’s not gonna move fast.” The same was true for understanding other math connections, such as when asked what they learned about speed. Eliciting responses showed *connections* students made along with corrections and guidance needed. In some instances, there was a *language interplay* in which the articulation provided students with the language needed to understand the *connections* between the *language of science* and what they were doing. For example, emphasis was placed on the units of measurement when they measured distance and time: “we were measuring millimeters…” or “empty bottle and…some water…they had equal time when they fell.” Students articulated using their own language and the language given by the instructor (Quinlan), as they pointed to patterns or connections they made.

### Reciprocal vulnerability

Student questions led the roller coaster engineer to *abstract from memories* of his childhood. When Mariah asked “But what made you want to, like, make roller coasters, and how long does the instructions take to make them,” Mr. Grady responded with the following:

Great question, so… I’ll give you the short version of why I wanted to… design roller coasters. So, my family used to go to amusement parks every year as a kid. And I just remember making a lot of great memories at the amusement park. And so, I knew at a very early age that I wanted to help people make… great memories at the amusement park too. So, you know, I wanted to give that experience to other people. And when I was 9, I got a magazine that came in the mail. It had a roller coaster on the front, and I thought it was such a beautiful thing. So, it was a combination of… of… my own personal fascination with roller coasters, and then also wanting to give people an opportunity to make memories. And so that’s why… I wanted to be a roller coaster engineer. In terms of how long it takes to make the instructions, it’s about a year and a half, about a year and a half.

Students themselves were *vulnerable* with the experiences they shared, which also led the adults to *abstract from their experiences* or *elaborate* with similar experiences. For example, students talked about their experiences, the thrills, and scariness of the “stomach drop,” in which the engineer indicated that he did not know the science behind it because he never studied it, but that it was his favorite part of the ride experience. In other ways, their *vulnerability was encouraged* by letting students know that their drawings did not have to be perfect or the engineer telling them:

Do not be afraid to experiment with things, try different things, you know. And if things do not work, that is normal. So, things not working the first time is a very normal experience as an engineer. So, a lot of times, sometimes kids get discouraged. Oh, my thing does not work. Well, you know. Things usually do not work on the first try, and that’s okay. That’s part of the fun (Mr. Grady, Week 3).

*Reciprocal vulnerability* was an important part of community between the adults and the children. Areas of overlap revealed an intentionality or goal to *engage family* or *develop rapport*. This involved Ms. Williams (guest teacher), who was a part of the community. She created interactive Kahoot activities or questions framed as “Would you rather this, or would you rather that?” The questions were related to food or places they wanted to go and were designed to build rapport. In drawing students out, she noted that students could respond even if they did not have their cameras on. In response to whether she would prefer to live underwater or on the moon, Halle noted: “on the moon because you cannot breathe underwater.” Part of this engagement involved the teacher sharing her own background as a former student of the first author and the HBCUs she graduated from. The students began by talking about what they had for breakfast and at one point the conversation segued into talking about chocolate when students were asked if they would prefer to watch the same movie every day or eat the same thing every day. *Engage family* was reciprocal in that the activities were included because of the feedback request from parents for more peer engagement opportunities.

#### Encouraging creating plans

This mainly reflected the goals of the instructor. Instructions were given beforehand to facilitate planning by parents/guardians and students in preparation for the next class. Class time required *microplanning* by the students and whoever was assisting them at home. For example, when measuring distance, students were asked to use what they had immediate access to, whether a tape measure or ruler. Parents with smaller children were directed to use Post-its that their children could count to measure distance. Children and/or parents or guardians needed to be involved in *creating plans* for successful participation. Most of all, successful participation was important for participation to happen. Small tasks required students to create *micro plans*. For example, students needed to identify something that they could roll during the forces and motion class. Quinlan (instructor) noted: “It could be anything … it could be a paper towel roller… It could be… A toy car, if you have one.” *Microplanning* was important and created a new situation for students, parents, and families involved where they could not actively participate if they did not plan for the new situation at hand, whether beforehand or during the activity.

Some micro plans included mental preparation to prepare for *spatial tasks* or *mental spatial planning,* where they first imagined and then carried out tasks, such as the predict-observe-explain activity. Students needed to prepare to first *imagine* what they were preparing for before they could predict it: “Do not do it yet. You’re gonna blow up your balloons. You’re gonna release it, but then I want you to draw everything that happened, to the air, and to the balloon.” The multiple steps required students to have a schema or planning knowledge for *planning* or for *spatial planning*.

At a different level, some *conceptual planning* was included to prepare students and their parents or guardians to carry out the bigger task of creating a roller coaster. The goal was to encourage them to create a roller coaster model. While mainly *conceptual planning* was used, the task required students to think about the bigger picture, to envision, and in some ways to think of it abstractly even when a lesson was designed to show them concrete examples of what others have done. They could only imagine it at this stage. In preparation, the instructor noted:

When we think about creating a roller coaster, there are many factors that go into it, to designing a roller coaster, and you can start from so many different places. Okay, so throughout, as we go through this, we are going to look at not just the science, but we are going to look at the engineering aspect as well. And so, we are so fortunate to have Mr. Grady to guide us and give us advice as well…When you are creating a roller coaster. Think about how high you want it. Think about the shape, the slope. Okay? Think about the number of hills…So we are going to use this week to plan…How high do you want it?

The entire class was focused on planning. The lesson proceeded by going through each variable, including the use of materials, so that participants saw video clips with people building a roller coaster using different materials. They also had the opportunity to ask Mr. Grady, the engineer, questions during this planning session.

Encouraging creating plans overlapped the most with community-foster individual voice, questions as connections, identify connections with science content and formal learning, and actual execution and explanation (see Figure 3). These connections are explored in separate categories in the next section.

*Questions as connections* were important for guiding students in creating *micro plans*: “Okay, what are you measuring with today? How are you measuring distance, how far it goes? Do you have a ruler? How are you measuring distance? You have a ruler? Do you have a tape measure? Oh, you have a tape measure, awesome!” Students were encouraged to ask their own questions to get the input of the roller coaster engineer. They were also prompted about the status of their *executions—*roller coaster building. While conceptualizing the variables and learning about science concepts, they were asked questions such as “What happens when the velocity changes?” This was important in helping them to *identify connections with science content and formal learning* and the building of their roller coaster.

#### Affirming assets

*Affirming assets* were reflected in different ways and came from different people. In response to viewing the video clip(s) interview(s) created for the lesson, Mariah pointed to the engineer’s *determination*:

I think it’s very cool how he went up to, like, the Maryland roller coaster people, and he was like, hey, here’s my resume. Now, we are gonna have, like, an interview, and they actually called him back. That’s, like…determination to, like, really work in it. That’s how you know he really wants to work in roller coasters.

Her response was affirmation for what was valuable to her—his determination. The engineer offered other *community assets*—*knowledge and historical assets*—when responding to what makes a roller coaster go: “There’s a bunch of mechanisms that make the roller coaster go, most of them powered by electrical circuits. Actually, the first engineer to create an electric-powered roller coaster was a Black man by the name of Granville T. Woods.” Introducing Granville T. Woods was affirmation for the *assets within the Black community* of learners. He was able to affirm his own assets when students asked whether he himself had built one or seen it built:

I was on the team who built … who made the instructions? So, I’ve never physically taken parts and put them together. I created the instructions for the builders to follow to make the coaster. I also chose parts to put pieces together, but I did not physically… you know, build the coaster with my hands.

He described his role as a part of a bigger team. He himself was an asset to the homeschooling communities Figure 4.

**Figure 4:**
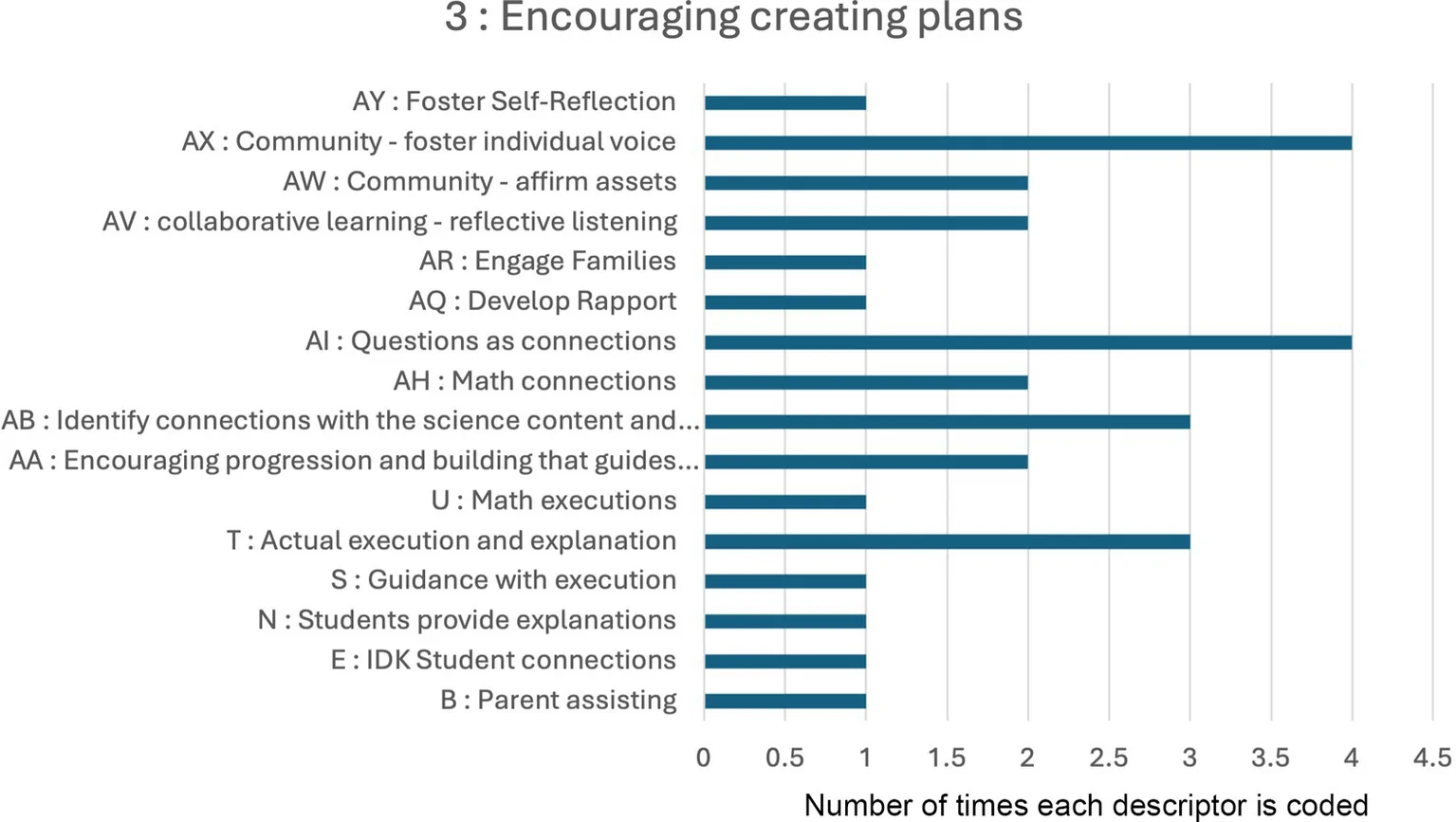
The frequency with which the code “encouraging creating plans” overlaps with other codes.

During the learning process, the science education researcher Dr. Likely contributed her own biology background to answer a question about feeling the stomach drop, and when Amani described an instance when she could not produce sound, Dr. Likely noted: “So the air going over your vocal cords wasn’t going the right way. That’s all. And because you now have other forces, like moving your body, you cannot push the air out in the same way. That’s all. Nothing was broken. It came back.” Quinlan, the instructor, applauded when the students provided new analogies to explain concepts they were discussing, such as Ella using the weather forecast as an analogy to explain what a prediction was. Students were assured in other ways, and *students’ assets* were important throughout the lessons both implicitly and explicitly.

During one of the activities, students discussed the stories they would use to create themes for their roller coasters, which gave them the opportunity to share their likes (*reciprocal vulnerability)*: Serenity indicated, “My favorite game is Fortnite, and I love language arts.” Ms. Williams’ response was affirming: “Oh, wow! Fortnite and Language Arts, very nice. I actually had one of my math groups called Fortnite in one of my classes, and I liked it. Years ago, I allowed some of my classes to name their own small groups, and one of them named it Fortnite, so that’s what I called my students all year.” They get to know each other—activities were designed to *develop rapport,* and the discussions ranged from talking about “breaking dancing is hard” to “eating good vegetables” to “pen, paper, and a judge,” where Ms. Williams asked, “Does anyone know who [she] is?” When Ms. Williams asked whether she was interested in working for a specific group of people, for families, or for children, she indicated, “I think I just want to work for families.” Thus, *engaging families* came up in more ways than one, not only as a goal for the instructor when asking parents for feedback, but as a possible future career. Ms. Williams indicated that she could not help but notice that the topic of food came up often, so she asked if they had anything delicious to eat.

#### Identifying connections with science content and formal learning

*Identifying connections with the science content and formal learning* overlapped with codes such as *foster individual voice*, *encourage creating plans, using science content to abstract from experience,* and *students provide explanations*. This code highlighted the intentions of the instructor. For example, Quinlan (instructor) used *questions* to elicit responses that would lead her to conclude the following from student discourse: “Okay, Newton summed up this in his third law. For every action, there’s an equal and opposite reaction.” Similarly, references such as “I want to say, in physics we describe the motion in different … we are using different words, right … sometimes we describe it as linear. Okay, what does linear mean?” The idea of making connections with science pointed to *integrating science into their lives versus integrating their lives into science.* The goal was science, but when it came to creating connections, the science was both the overarching goal and the secondary goal, such as: “Use what you have in your hand to create the motion, okay.” Access was important, and their surroundings were centered and used to understand the science. The idea of *using their surroundings to understand science versus using science to understand their surroundings* showed that while science was the overarching goal, the importance was in the connections that could be made, as if to say *if there was not a connection, there was not a science*. The instructor noted: “Yours traveled 77, wow, okay. So, when you are done, push it harder and tell me how far it traveled. Okay? Pushing it harder means you are giving it more force.” *Their experiences were centered as much as science was used to center their experiences*. The intention was to use science to plan, but the goals of the instructor positioned the science as both front and center and, at the same time, secondary to connecting with their lived experiences.

The sense of *community and fostering individual voice* was important in correcting perceptions rather than the instructor simply telling a student that her experiences were incorrect when it was what she imagined as her experiences:

Catherine Quinlan: What about the drop? Which one makes you feel heavier or lighter?

Janiyah: The drop makes me feel heavier because I’m going down. And going up makes me feel lighter, because I’m going up.

Catherine Quinlan: Okay, so here’s my question. Which one makes you feel like you are pressed against the… Which one makes you feel like you are pressed against the roller coaster versus you are really loose in the roller coaster?

Janiyah: When I go down, I feel pressed against, and when I go up, I feel really loose.

Catherine Quinlan: Okay. Alright, thank you. Anyone else? Thank you, Justice. Did anyone have a different experience? Adults, you can feel free to share your roller coaster experience, too. I see Ezekiel.

We see both community and individual voices and their abstractions from their experiences at play here, where the instructor called on the adults as well to share their experiences. Zina abstracted from her experience riding a bicycle to explain this feeling or phenomenon. The instructor used *repetition* in describing the phenomenon with repeated statements such as:

One of the reasons we often feel heavier when we are going up is because we are going up against gravity, and it’s pushing against us, okay? And when you are falling down, sometimes you feel like you are about to bounce off of your seat. I do not know how many, you know, which is why you have… which is why you have those straps, right? So, let us look at these roller coasters here.

Zina *abstracted from her experience*: “When you are going… When you are going up, it’s going against gravity, like when you ride your bike. When you ride your bike up the hill, it’s harder. But when you ride your bike down the hill, it’s easier.” As always, throughout the lesson, the instructor is explicit about connecting this lesson to a larger goal to *encourage creating plans* by looking at “energy in a roller coaster,” noting the different kinds of energy and asking students to consider: “What kinds of things do you think will increase the potential for motion, giving your roller coaster more potential for movement?” The *connections to science*, *abstracting from their experiences,* and using their *individual voice* within the *community* were all part of a larger goal to empower individuals or families for execution later.

#### Connections and proximity to Black children and to blackness

While the goal of this entire research centers around *connections and proximity to Black children and to Blackness*, the importance of creating this code pointed to explicit narratives about this and opportunities for additional insights. This code ranked thirteenth in terms of what was salient to the researcher, and overlapped the most with *reciprocal vulnerability*, *questions as connections, connections with engineer,* and *articulate affirming counternarratives.* The excitement and joy at seeing the children was evident among the researchers, as seen in the instructor’s comment: “But of course, as usual, I do love to see those beautiful little faces there. It’s so awesome seeing you guys.” Ms. Williams shared about her experiences attending HBCUs in response to students. The joy was reciprocated by parents/guardians and children, with a great deal of energy and participation throughout. Big and small *questions* became *connectors,* as the engineer anticipated them with the comment: “Cannot wait to answer your questions. At least do my best.” No question was treated as minor; whether students asked, “what kinds of materials do you use for, like a roller coaster, if you were making one?,” which gave the engineer an opportunity to share how he used his kitchen table and chairs in response to being asked, “what twist and stable material would you use?” Again, the *vulnerability* displayed by the children was *reciprocated* such that the engineer shared memories from childhood:

I remember when I was 4, stepping… sitting inside of a roller coaster felt huge. It felt like I was swallowed up by the roller coaster, but then when I became an adult, now sometimes I struggle to fit inside. My legs are too long, and I’m having to adjust and everything, and so it really… Depends on the person who’s riding it, on how big it feels. (Mr. Grady, Engineer).

While other examples shared by the engineer might be considered more vulnerable, here we see the engineer reach into his own personal experiences to relate moments that could be considered an embarrassment for others—to be too small and told you cannot get onto a ride or to be too big for the ride. Ms. Williams also *reciprocated* by reaching back in time to describe other memories:

When you said neck bones, you got my southern heart. If I have not said this in here yet, I’m pescatarian, so I do not eat meat. I only eat seafood, so, you know, fish, shrimp, things like that, but… when I was eating meat, a neck bone, my mom could cook a neck bone down. We put some rice, or, you know, anything goes with neck bones, but I see you guys, young people, know about the neck bone (Ms. Williams, Guest teacher).

This was treated as a moment of *reciprocal vulnerability* and specific to proximity to Blackness because these were not considered conversations about food someone would openly divulge with everyone in Western culture, but rather became a bond within this community.

### Part 2. Funds of knowledge

A Funds of Knowledge framework elucidated the specific ways this Roller coaster Physics curriculum invited Black elementary and middle school students to showcase their funds of knowledge as these relate to STEM. From the outset, students were invited by the instructor (Quinlan) to view an interview with Mr. Grady, a Black roller coaster engineer, and to ask Mr. Grady questions about his experiences and expertise as these relate to their own experiences and existing knowledge. Instructors were creating a community of learners by orienting the learner to experiences that are authentic and relatable, honoring students’ funds of knowledge, centering learners’ experiences and wonderings, framing responses to children’s experiences and inquiries with STEM language, and showing enthusiasm and interest in this physics topic. Two themes prevailed when discussing findings with co-researchers: *Community of Learners* and *STEM Language.* Below, we focus on salient codes under each theme.

#### Community of learners

The theme *Community of Learners* emerged from the following codes: *Assisting with learning (family/adults); Connecting to/between learners; Showing interest/enthusiasm.* Over the course of eight online sessions, where children across elementary and middle grades in homeschool settings were invited to explore roller coaster physics, children were assisted with their learning by family members and adults who were present; children and adults made connections to and between learners; and children and adults demonstrated interest and enthusiasm through verbalizations, facial expressions, body language, and typed responses via chatbox. Below, we share excerpts as evidence illustrating how this online community of learners engaged in co-learning as they explored roller coaster physics.

### Assisting with learning (family/adults)

Data from weekly transcripts showed how adults and family members assisted children by: asking questions of Dr. Quinlan and Mr. Grady, communicating with session leaders to seek support for technical issues, talking directly to session leaders to ensure their children had a chance to share, asking the children prompting questions, encouraging active participation, reminding children how to utilize their materials and resources, directing children on how to complete a task, reminding siblings to be agentive and fair in their learning, expressing enthusiasm for and active engagement in the curriculum, and reminding children of learner behaviors (e.g., *show, look, listen*). See Table 4 for transcript excerpts from across the 8 weeks of online sessions.

**Table 4:** Transcript excerpts with family assisting with learning.

Excerpts from weeks 1 to 3	Excerpts from Weeks 3 to 8
*Wk 1* Well, I do not know how to raise my electronic hand, but my kids wanted to share. *Wk 1* I think she’s saying, “How does it go? Because she was asking me earlier. *Wk 1* Oh, did you hear that? Say it loud so they can hear you. *Wk 2* What does it mean? Are you feeling strong? Hurry, say what you think is *predicted*. *Wk 2* Show her the picture. *Wk 2* What direction was the air going? *Wk 2* Draw with arrows, that kind of motion. *Wk 2* It’s in your book bag, on your desk. *Wk 2* What did you want to share? She’s calling on you. *Wk 2* Just to clarify, sorry, homework was watching the videos from the previous week, and then the Khan Academy thing? *Wk 3* Wait, wait, she can speak for herself. [pseudonym], do you want to talk about your favorite Pokemon story, or your story? *Wk 3* Yeah, we were watching it, and then, and then we were talking, so I wasn’t sure if we are supposed to watch it with you or now by ourselves. *Wk 3: I think he was talking about the induction engines;* we do not know how to…recreate that… Can we recreate it in our project with magnets?. Because [pseudonym] wants her ride to be slow. She said, I do not want all that loop-de-loop stuff. She said, I want it slow, but maybe if we could do magnets, it can pull it slowly for her. *Wk 3* Also, I was gonna… I wanted to share, because we were doing a little research on roller coasters. And on YouTube, Hersheypark has, like, there’s a guy who went to Hersheypark and rode every single roller coaster and recorded it from the front seat, so it looks like you are on the roller coaster, and it gives a really good view of the track, and the twist, and the turns, and all the things. And he has a bunch of different types of roller coasters there, so it’s 20 min of, like, just roller coasters, so if anybody wants any ideas on what to build or how to build, that’s a really cool thing to watch…Okay, I’ll find it and send it to you.	*Wk 3* What are you doing here? You’re very busy. What are you making? *Wk 3* I had a question. I’m [pseudonym]'s mom. I… We’re a curious crowd over here, so… I wanted to know, first of all, what do you teach? Do you teach physics? *Wk 4* Show the inside. [referring to a backroller the child was showing on screen] *Wk 5: You did not raise your hand;* do you want to build one? *Wk 5* You can keep changing it, honey. *Wk 5* Can you just click on stop share? Hold on. Alright, hold on, baby. Oh, here we go, okay. Okay, how do I get out of this? *Wk 5* You are gonna build a roller coaster… What do you want it to look like? Where’s your notebook?. Where’s your notebook where you drew it? It was on there… What are you gonna use to build? *Wk 6* I really, really want to sign up for the science fair. I do not really… I’m on the Eastern Shore of Maryland, so it’s gonna be a little bit of a trek for us to go over there, and I’m fine with that, but I was just curious. I do not really know much about how to do, like, the science project. I know [pseudonym]'s question is, like, how do birds fly, and how do they use their feathers to fly? So, that’s kind of where he wants to go with it, but do we… Is there any support… *Wk 7* No. Sit down [pseudonym]. Where did you draw? It’s in your red notebook. Where is it? *Wk 7* What’s the story that you are trying to tell?. The creation and the fall. *Wk 7* Do you wanna say something? *Wk 8* Can somebody help me? My daughter just said she does not know what to do, and I do not know what to do either…[pseudonym], come with your… It’s right here, honey… Sit over here, so you guys can do it. Take that off your head, honey. *Wk 8 [older sibling]* If he’s on iPad, he should be able to split his screen… She can split screen on iPad by double-tapping the home button and dragging Zoom and Kahoot together, and it will split the screen. *Wk 8* Something’s going on with ours; it’s answering the question before…

Statements from family members and adults present showcased the sharing and building of funds of knowledge, including *caregiving, technology skills, storytelling,* and *resourcefulness.*

### Connecting to/between learners

Prevalent in the data (e.g., weekly transcript documents), participants of all ages and experience levels connected with other participants as co-learners. As evidenced in data from the Week 1 session, centering children’s interests and experiences in the curriculum involved developing connections and learning from children from the very beginning. In this study, session leaders were schema-building by first learning about what children knew about roller coasters, from observation and/or personal experience.

Connections to and between learners were evidenced through verbalizations, facial expressions, body language, and chat responses (not seen in the transcripts but read aloud by session leaders). These connections were evidenced at particular points during the online sessions, including: verbally greeting one another; stating one another’s names; sharing experiences, interests, and ideas; articulating personal connections; verbalizing recognition (e.g., *I see your beautiful face*); checking for access (e.g., *Did I see your hand? Can you hear me? Everybody set?*); inviting learners to co-teach (e.g., *You’re gonna help me, I let you all take the lead based on your interests*); engaging in joint attention (e.g., *You know, I see that too, Just imagine…*); expressing appreciation (*I felt like we were learning so much from your questions*); sharing questions and concerns; inviting responses (e.g., *I saw your hand, You had a question?*); reassuring children (e.g., *You’re safe*); making comparisons and building schema (e.g., *Just like…*); repeating/amplifying what has been said; offering choices; checking for understanding/connection (e.g., *Does that make sense?*); stating the desire to share (e.g., *I had a question, I have something to say, Can I ask you another question?*); confirming understanding; validating (e.g., *That’s true, That was your experience and I believe you, You all had a lot of amazing questions, Trust me I feel the same way*); attempting to understand; (e.g., *So…, Yes, okay*); and attempting to make a connection (*You know what’s interesting?*).

#### STEM language

STEM language was central to this Roller Coaster Physics curriculum, and the researchers were particularly interested in how children were taking up and utilizing STEM language. Codes under this emergent theme are listed below, and the code relating to children’s articulations will be discussed in detail.

### Aligning response with STEM learning objective

At the beginning of the online sessions, children shared what they already knew about roller coasters based on experience (e.g., riding a roller coaster) and/or observation (e.g., seeing roller coasters in a movie or show, reading about roller coasters in a book, watching an informational video on roller coasters). From the start, children used STEM-informed words and phrases such as “shaky,” “how to make them move,” “have them stop,” “loop-de-loops,” “twists,” “turns,” “drops,” “would go down, up, to the side, and sometimes you would go fully up, and you’d be like, why is it stopping? And then soon, it just goes down until you drop,” “speed,” “height,” “I like the ones that go high,” “I go around in these directions and just have fun,” “Some are fast, some are slow, some go up to the clouds, some do not.”

The children also noted that they valued Black leaders in STEM. Musa noted, “I would say that I feel like it’s really cool that you’ll be able to, like, make roller coasters, because I feel like there’s not, like, a lot of Black males that make roller coasters.” Mariah noted, “I think it’s very cool how he went up to, like, the roller coaster people, and he was like, ‘Hey, here’s my resume. Now, we are gonna have, like, an interview.’ And they actually called him back. That’s, like, their determination to, like, really work in it. That’s how you know he really wants to work on roller coasters.”

The questions children shared demonstrated both their existing schema related to age, experience, and overall interest. For example, Xander inquired, “I wonder how roller coasters, like, stay in their spot, so when they go upside down, they do not just fall right down.” Mr. Grady asked, “How do you make a roller coaster spin?” Children continued to ask questions such as, “How do you make roller coasters?” and wanted to know who all were involved in the building. Amani inquired, “How do, like, wooden roller coasters work? Like, how would that stay up? It seems like that’s, like, extremely dangerous for roller coasters to be wooden, but I believe there’s still one at Six Flags, unless they tore it down, and a few others.” Building on one another’s questions, children asked, “Why’d they make them out of wood, of all things? They could have made them out of a lot of stuff, but why’d they choose wood?” and “How does the roller coaster stay stable? Is there a test, like, to make sure it’s safe?” Mariah was interested in the engineer’s motivation and asked, “What made you want to, like, make roller coasters, and how long do the instructions take to make them?” Someone built on this question and inquired, “How many roller coasters do you think are built a year?” Children were increasingly interested in the construction and experience of the roller coaster and asked, “What are they made of, and how are, like, how do they feel inside when you ride them?” as well as “How big can a roller coaster be, and what is physics?”

Drawing on her own experience, Zina asked, “Why do roller coasters stop in the middle of the ride?” Vividly recalling his experience, Maurice stated, “Winter roller coasters are always safe. But I do not think all of them are, because the one that I went on one time was so short that, if my dad had been like 2 inches taller, he would have hit his head. Because I feel like people were probably shorter back when they made that. Because that was, like, a really old one. I think it was, like, more than a 100 years old.” Remembering her experience, Ebony asked, “Why do roller coasters always make you, like… why when they go down, they always just make you feel weird?” Xander connected to this by stating that they did not like the feeling and that they got a similar feeling on a swing. “Like, when I go on a swing, and it goes super high, my stomach feels weird.” Amani continued to explore the ways that roller coasters can make a person feel: “This might be different, but, like… I remember one time when I was on one of those, like, drop rides where it goes up and down and up and down. And I got… this was my first time going on one of those types of rides, so I tried to scream going down, and I do not know why, but, like, I physically could not; like, nothing was coming out. I do not know why, but it wasn’t coming out.” Xander related to this by sharing: “One time, I was on a huge slide. It kind of like drops, and… I was on the back of the slide. And it was, like, a full ride, and I had my friends. And I went down. I was on the back, and when I went down, it immediately just dropped down, and, and it, like, my stomach was, like, up. It was high-pitched.”

Returning to the properties of the roller coasters, Janiyah asked, “What’s, like… the height limit for Maryland on roller coasters?” and “What’s the tallest roller coaster you have been on?” Adding to these questions about height, Deshawn drew upon his experience and stated, “I wanna know… when roller coasters, like, have a loop-de-loop… One time, like, I was on this, like, ride, at this, like, fair, and it was just, like, a pure circle. And I wanted to know, like, roller coasters are a circle. How do they function? Because it was just like a roller coaster, like this. And the roller coaster just goes in a circle.” Continuing to build, Sarah asked, “How do you think the roller coasters evolved over the years, and how much electricity or power is used to make one roller coaster work?” Wonder Ride followed up, drawing on his extensive experience: “I’ve been on a lot of tall roller coasters, but what I’m wondering is how it survives the air force without malfunctioning.” Across the weeks, additional physics terms were introduced and explored, and the children continued to demonstrate the development of conceptual knowledge.

### Part 3. Benefits of including the Black lived experiences and knowledge

#### Overall summary using Schema theory and Funds of Knowledge frameworks

The benefits of centering children’s interests while explicitly incorporating their Black heritage into the STEM curricula led to questions as connections, seeking community, reciprocal vulnerability, and fostering individual voice. Shared experiences provided space for questions to become connections. The students were encouraged to ask Mr. Grady questions without prompts, following their own interests in roller coasters. Seeking community was a common goal of the students, which led to engaging families as a part of the community of learners. The students were able to create plans, which led to explanations in real-time alongside their siblings and parents or guardians, plus the educators and roller coaster engineer. Within the all-Black community of learners, the opportunities to engage in conversation fostered individual voice and expanded STEM language. For instance, the conversations during each lesson used explicit science vocabulary and explanations of various physics phenomena in the students’ own words. Along those lines, the home and community contexts served as a foundational mechanism for connecting scientific knowledge in ways that are culturally relevant and grounded in community. These conversations happened amongst a broad community of learners that included siblings, parents, educators, and the roller coaster engineer. These elements affirmed reciprocal vulnerability, assets, and connections with science content. Additionally, the findings highlighted the importance of cultivating community toward conversations for science connections. The research affirmed that creating learning environments in which Black students could thrive academically while also affirming their cultural identities were essential for culturally responsive and inclusive pedagogical approaches.

#### Overall themes emerging from both frameworks

The overall themes emerging from both frameworks point to the benefits of using the lived experiences, narratives, cultural knowledge, and heritage to guide the creation and implementation of curricula.

*Accessibility.* Schema theory, integrated with social, emotional, and cultural categories, highlighted the processes the community was engaged in. These processes signified student engagement, indicating that the content was accessible enough to encourage students to use their own assets and funds of knowledge to provide explanations displayed in individual and communal voice. This perspective was confirmed by the engineer’s reflective interview after the lessons. When asked about his observation and his experience in the two sessions he attended, he noted the following:

Mr. Grady (Engineer): So, my takeaway was definitely. Okay kids, here, this is something you can do. This is a topic that belongs to you, and you should feel at home in this subject matter. So, I feel like that was the foundation. I feel that was the tone that was set from the very beginning. Okay everybody, you belong here. Everything we are getting ready to talk about, you belong here. And so, making it accessible in that way through narrative. I’m sure it just opened up their minds and hearts even more in terms of their confidence. Then, you know, once their confidence is up, now they can really learn because the barriers aren’t there.

Accessibility came from belonging in the content and belonging in the process and through recognition and culturally affirming narratives, practices, and processes for engagement.

*Embedding.* A second theme was the *embedding* of the community and home contexts within the learning content and process. This was also supported by the situation or state of affairs that the Funds of Knowledge framework pointed to. The ability to form connections between the science content and storytelling was displayed in students’ use of STEM language. Families assisted with learning and were engaged in the process. In his interview, the engineer explicitly pointed to “communal pedagogy” as “a real strength. That was really excellent…cause it’s almost like teaching through, by village. It’s like village instruction.” The impact on the children during the evaluative focus group after the program ended also supported this perspective. Xander stated: “So I know how much gas my mom needs for however many miles my mom is gonna go.” He then went on to say: “So I wanted to add something. I really like this class, and the reason why I like it is that I like the people, and I like Ms. Williams, and everybody’s personality…[I was surprised] that Ms. Williams was a teacher!!!” The unusual nature of the interactions in formalized science learning was supported by explicit connections students made, and children’s reaction to finding out that about the teacher.

*Empowerment and Joy. Showing interest* and *enthusiasm*, student voice and vulnerability pointed to a level of empowerment and joy, which was evidenced by the level of engagement. This was supported not only by the connections made but also by the *resourcefulness* and *rapport,* among others. These findings were supported by students’ explicit feedback when asked what they liked about it: “I learned more than I thought I would,” and later repeated, “I liked basically everything” (Kam). Another said, “Actually, the class was very fun, because I got to learn some stuff” (Lauren). Another stated: “And the teachers, the teachers were really nice and broke everything down, so I enjoyed that.” The enjoyment and empowerment were embedded in the learning, as seen in Xander’s statement: “So what I learned was, I learned how to calculate time on a car, like, something fast. *You mean speed?* (Mom corrected). …I mean speed, which I did not even know how to do,” Then later added: “My favorite was calculat[ing] time.” (Xander). Several indicated their sadness at the end: “I’m sad at the last week.” Another wrote in the chat, “Noooooo I am so sad” (Zana) along with the crying emoji. Another indicated, “But I loved, like, he [the engineer] could answer all my questions that I asked him.” (Sarah) Another noted: “What I liked about… what I thought was valuable was learning about his life and how he… he got his job” (Xander). When asked for advice or what can be changed, one student noted: “Nothing was really needed; it was pretty perfect” (younger sister). “For me, my advice would probably just be to keep being the great teachers that you are” (Sarah). The joy was evident on the faces of the children. There was smiling and laughter during implementation, or sadness that it was ending.

## Discussion

The findings show that including the lived experiences and narratives of Black heritage and Black people contributed to knowledge and processes that supported building schemas and learners’ social, emotional, and cultural connections, as well as learners’ funds of knowledge. The frameworks complemented each other such that using Funds of Knowledge as a framework supported the findings which showed connections between individual schemas and schemas which support social, emotional, and cultural connections and vice versa.

### Part 1. Schema theory as a framework

While schema theory may support individual schema construction, pattern recognition, abstractions, and repetitions (Marshall, 1995), the dialogue data from the community quickly revealed the importance of considering social schemas. The American Psychological Association notes that “social schemata often underlie behavior of the person acting within group contexts, particularly larger groups or societal contexts” (American Psychological Association, n.d.). The dialogue evidence shows connections with social, emotional, and cultural considerations, which align with Markowitz and Bouffard's (2020) “practices to develop the social, emotional, and cultural lens” (p. 28), which led to these practices being used to code the data. Markowitz and Bouffard note that the goal of these practices was: “1. A safe, brave, and supportive learning environment; 2. Equity in teaching and learning; 3. Resilience and sense of optimism; 4. Academic success; 5. Responsibility for the greater good” (p. 28). The dialogue and interaction provided evidence of a safe environment and implicated a shared goal of everyone who participated.

#### Connections and proximity to Blackness

While it might be surprising to the reader that *connections and proximity to Blackness* ranked low in abundance, this is not because of its lack of significance; connections and proximity were present throughout, both explicitly and implicitly. Therefore, it was important to ask questions about the benefits. All the positive outcomes and benefits are connected to inclusion and predispositions of everyone within this Black community—the Black science education researcher, the Black male engineer, the Black teacher, the Black parents, Black children, and Black guardians, and it is this community which led to the abundance of the outcomes. While this research only addressed the dialogues during implementation, it is important to mention that, in the debriefing, the African American engineer commented to the effect that he felt that the nature of the community itself had removed barriers to participating and communicating for the children. This can be likened to Syed et al.’s (2012) findings, which show that the White students who were always in the “high contact” groups with their mentor indicated no preference for whether their mentors had a background that matched theirs. This was unlike the students of color who were mainly in the “no contact” or “low contact” groups and indicated higher preferences for mentors with matched backgrounds. A lack of feelings of belonging was accompanied by an expression of preference for matched backgrounds. Students’ explicit expressions of joy about the programs and how much they loved the “instructors,” their sadness that it was ending, and wishes that they could do this again confirm their feelings of belonging in the community.

### Part 2. Funds of knowledge as a framework

While funds of knowledge pointed to the broader strokes, schema theory pointed to the finer strokes, which point to more detailed funds of knowledge. Both sets of perspectives were considered warranted and provided insights. The significance of pointing out the results of both sets of analyses places emphasis on the role of multiple perspectives as opposed to one correct answer or one way, while highlighting the significance of distinguishing analyses that point to broader or general outcomes versus the finer strokes which result from complementary methods. Schema connections show that the *community of learners* included family, parents, teachers, and learners, along with the positionality of science education researchers who implemented or participated, which were central to fostering individual voice, reciprocal vulnerability, affirming assets, and engaging families. The funds of knowledge findings, which pointed to the promotion of STEM language, were supported by STEM learning objectives and a schema-building environment that encouraged creating plans and connections with the science content and formal learning. The extensive dialogues and connections have important implications for the extent of pattern recognition (identification knowledge) which students can elaborate on (elaboration knowledge), and the subsequent skills and planning knowledge they display for schema development. The exact nature of the knowledge types and schema knowledge that led to their execution knowledge needs to be further explored.

#### Funds of knowledge: caution, limitations, and implications

Pragmatic curricula design and methodologies informed by best practices in science education pedagogy were used to create and implement the curricula, while the funds of knowledge were only used to analyze the outcomes. Namely, the use of Black students’ cultural knowledge and heritage knowledge conveyed additional benefits that supported students’ funds of knowledge. Teaching diverse learners involves deeply understanding children’s and families’ funds of knowledge, including their occupational expertise, language practices, and cultural experiences. As the studies reviewed by Rios-Aguilar et al. (2011) indicate, when teachers leverage children’s and families’ funds of knowledge in formal school experiences, we develop a more meaningful curriculum that honors family expertise, connects content to existing interests and expertise to build schema, integrates attention to children’s thinking, and ultimately creates learning environments that serve diverse students.

Funds of Knowledge as a framework provides a general overview of the broader strokes of the study. In studies that seek to amplify the knowledges and expertise of socioeconomically, linguistically, and racially diverse communities, Funds of Knowledge has often been utilized as a theoretical framework to inform methodology. After decades of education researchers implementing FoK in partnership with under-represented communities, founders of the framework have urged researchers using FoK to critically examine frameworks that (a) have informed the Funds of Knowledge framework, (b) are informed by the Funds of Knowledge framework, and (c) have sometimes been conflated with Funds of Knowledge. Critiquing the conceptualization and use of FoK more broadly, Rios-Aguilar et al. (2011) remind educators to recognize “the resources that students bring to the classrooms and that challenge dominant schooling practice” (p. 171). They remain adamant that recognizing under-represented communities’ funds of knowledge is not enough; if American schools want to honor and include the historical and cultural knowledge of under-represented communities, educators need to create learning environments that “activate/mobilize” under-represented communities’ social and cultural capital. Furthermore, the authors argue that educators should continue to confront the *(mis)*recognition of expertise that under-represented populations bring to school (p. 176).

Yosso (2005) has leveraged Critical Race Theory to remind educators of the historical erasure of contributions from Communities of Color, as well as to illuminate the community cultural wealth of Communities of Color. While Funds of Knowledge lends a valuable framework and a methodological approach that positions teachers and researchers as co-learners who all bring valuable knowledge and skills to the learning community, coupling FoK with critical frameworks such as CRT enables educators to *move beyond recognizing* the brilliance of Black children—to actively transform policy, research, and practice toward socially just futures. In the current sociopolitical climate—where we are witnessing a new wave of policies intended to misrepresent and erase the societal contributions of Black Americans—we seek to engage educators in interdisciplinary and transformative practices that amplify the stories of Black educators and Black children who are co-learning within communities that invite everyone to share their linguistic brilliance, scientific ingenuity, and mathematical genius.

### Part 3. Benefits supported by critical perspectives in science education

Following Tan et al. (2013) in rethinking an equitable science experience for students through asset-based curriculum and assessment, this research incorporated assessments that were inclusive of relevant communal practices and supported sense-making opportunities through expanded evaluation tools that support and empower all student voices through social processes. Specifically, the activity and assessment allowed students to not only create a model but also to represent conditions that would affect the model given a specific phenomenon. Implications for the broadening of curriculum include expanding culturally centered and sustaining assessment opportunities. Students could reorder power dynamics in the curriculum/learning material and were not constrained by materials or instructions. As such, agency rested with the students to inform them how they were modeling.

### Implications for motivated thinking in curriculum development

Curricula that promote Blackness and Black assets require additional predispositions and pragmatic methodologies that consider the broader goals and motivated thinking of those involved, leading to different considerations and questions asked (Quinlan, 2021; Quinlan, 2024a, 2024b; Quinlan and Farina, 2025). Molden and Higgins (2005) distinguish between two motivational orientations: a promotion focus, or avoiding errors of omission, and a prevention focus, or avoiding errors of commission. Molden and Higgins (2005) note that “preferences for eager judgment strategies, shown by those with promotion concerns versus preferences for vigilant strategies, shown by those with prevention concerns, alter many basic cognitive processes during reasoning” (p. 310). Among these cognitive processes altered were “an emphasis on fast versus accurate processing of information” and “knowledge activation and recall” (p. 310). Molden and Higgins note that one’s motivated thinking may influence a goal for accuracy or closure and “how much activation, evaluation, or explanation in fact, occurs” (p. 303). This is reflected in research on outcomes from altering curricula for goals that do not promote the assets of Black children but might consequently accentuate what Black women science education researchers consider deficit perspectives (Smith-Mutegi et al., 2025).

Blackness in science curricula shows up as indigestible when it highlights Black scientists or other content unrelated to the concepts under study, without pedagogical considerations, leaving the learner and teacher with the impression that Blackness in science is only ceremonial, not meaningful, and has no place in the curricula. Coles notes the following perspectives, which align with motivations that drive this work:

I gained insights into the ways Blackness was positioned as indigestible to curriculum through the overabundance of white teachers, which implicitly signaled to me that Black people cannot be disseminators of knowledge as they are counter (to) curriculum. This was reinforced by the ways my predominantly Black schools were considered to be inherently anti-intellectual. The whiteness of curriculum led me to think that I—Blackness personified—was out of touch with or outside curriculum. I was schooled in this logic: the idea that Blackness and Black people are outsiders on intellectual matters. That logic is an ideological tool of white supremacy. Unfortunately, curriculum studies are tethered to such ideological falsehoods, which must be disrupted. Now. (Ohito and Coles, 2021, p. 20).

Therefore, Quinlan (2021, 2024a, 2024b) has made it her goal to develop and use her expertise in science education pedagogies to both create curricula with content and pedagogical considerations on her own terms, and to highlight the assets of Black people and Black children (Quinlan, 2019, 2021, 2024a, 2024b). Rather than shying away from alternative perspectives, by grounding this approach in the literature, this study seeks to explore and highlight other perspectives and to ascertain the strengths and limitations of alternative perspectives, based on the predispositions of the researchers. In prior research, Quinlan (2024a, 2024b) found that the funds of knowledge was not specific enough or adequate as a means to an end, and did not address the science capital of Black people, but in this research wondered what funds of knowledge might reveal if used to assess outcomes, after using pragmatic methodologies to achieve the desired goal of situating Black heritage, lived experiences, and narratives in the science curricula. Foundational researchers on Funds of Knowledge (Llopart and Esteban-Guitart, 2018; Rios-Aguilar et al., 2011) encouraged a reimagining of its use in other contexts so as not to limit its use and so that it continues to evolve.

### Future directions

#### Family engagement

Several critical directions for future research on homeschooling, family engagement, and science identity development can be explored. Longitudinal research is needed to trace how parent–child science interactions and the resulting identity constructions evolve, particularly as children develop greater independence and as parents’ pedagogical approaches adapt to increasingly diverse scientific content (Calabrese Barton and Tan, 2018). Comparative research examining science identity formation across diverse homeschooling contexts, including secular versus religious homeschools, classical versus unschooling approaches, and families with varying socioeconomic and cultural backgrounds, would emphasize how pedagogical philosophies and value systems shape the nature and quality of science engagement (Apple, 2000; Kunzman and Gaither, 2013). Research should investigate how homeschool families access and integrate external scientific communities, including online resources, museum programs, and mentorship relationships, to understand how these connections might supplement familial recognition and provide broader networks for science identity development (Nasir and Cooks, 2009). This would further advance theoretical understanding of science identity through family engagements and provide practical insights for supporting families through homeschooling and traditional schooling in cultivating children’s engagement with science.

#### Other future directions

What could be construed as limitations, this study views as opportunities for future growth, given that research specific to curricula development using pragmatic methodologies points to the future of including the lived experiences and narratives of Black heritage and Black people. The researchers acknowledge that more popular and accepted research literature exists but choose to focus their energy on generating robust findings rather than theorizing. While the findings naturally included implications for literacy and family engagement, due to the complexity of this research, the focus of the study was on what could be systematically proven using the frameworks and the data.

This research provided an opportunity for both theoretical frameworks to support each other, where schema theory could be integrated to support funds of knowledge and where funds of knowledge could be integrated fully to support schema theory. The researchers view this as an opportunity to continue to explore these methodological significances using these and other frameworks in future research. Further discussion to integrate these frameworks could enrich the study and might lead to different or more integrated findings. This study generated a large amount of data, and future reinterpretation could provide a different perspective. Furthermore, other research collaborators might use different frameworks which provide a different emphasis and lead to alternate findings. The next step is to test this implementation or lessons in the school classroom setting to understand the extent to which the approach and/or content is transferable to a different context.

## Conclusion

There are important distinctions between frameworks that analyze and explain, and those which create products that support social and emotional learning, funds of knowledge, disciplined literacy, and family engagement. A framework that is valuable for explaining may not be sufficient to achieve the same ends it supports. This is especially true for STEM curricula products that situate Black and Indigenous cultural and historical knowledge. The predispositions of the researchers and curricula design expertise specific to STEM are central, and so is knowledge that supports the development of social, cultural, and cognitive schemas. Pragmatic methodologies, approaches, and ways of thinking and being are central to pointing to the future when abstraction from the past is not sufficient to propel this work forward.

## Data Availability

The datasets presented in this article are not readily available because the data consist of video recordings of minors. Requests to access the datasets should be directed to cquinlan@nccu.edu.
