# Supplementary material for: Building schemas through the lived experiences of an African American roller coaster engineer: Interdisciplinary analyses of students’ dialogues in a physics unit
Source: Front Sociol. 2026 Oct 9;11:1859066. doi: 10.3389/fsoc.2026.1859066 (PMC13690534; doi:10.3389/fsoc.2026.1859066)
Supplement: Supplementary file 1 [file Supplementary_file_1.DOCX]

**Appendix A**

**Connections to the NGSS and CCS**

| Next Generation Science Standards (NGSS) | | | |
| --- | --- | --- | --- |
| *Performance Indicators*   - Plan an investigation to provide evidence that the change in an object’s motion depends on the sum of the forces on the object and the mass of the object. - Analyze data to support the claim that Newton’s second law of motion describes the mathematical relationship among the net force on a macroscopic object, its mass, its acceleration. - Planning and Carrying Out Investigations to compare the effects of different strengths or different directions of pushes and pulls | | | |
| *Science and Engineering Practices*   - Planning and Carrying Out Investigations - Analyzing and Interpreting Data - Constructing Explanations and Designing Solutions   *Connections to Nature of Science*   - Science Models, Laws, Mechanisms, and Theories Explain Natural Phenomena - Science as Human Endeavor, including ex. Men and women from all cultures and backgrounds choose careers as scientist and engineers. - Science Addresses Questions About the Natural and Material World. | *Disciplinary Core Ideas*   - Motion and Stability: Forces and Interactions - Forces and Motion - Types of Interactions - Relationship between Energy and Forces - Definitions of Energy - Conservation of Energy and Energy Transfer - Defining Engineering Problems - Defining and Delimiting an Engineering Problem - Developing Possible Solutions | | *Crosscutting Concepts*   - Cause and Effect - Systems and System Models - Energy and Matter - Stability and change - Connections to Engineering, Technology, and Applications of Science - Influence of Science, Engineering, and Technology on Society and the Natural World |
| Common Core Standards Connections (CCSC) | | | |
| ELA/Literacy   - Follow precisely a multistep procedure when carrying out experiments, taking measurements, or performing technical tasks - Conduct short research projects to answer a question (including self-generated question), drawing on several sources and generating additional related focused questions that allow for multiple avenues of exploration. - Describe measurable attributes of objects, such as length or weight. Describe several measurable attributes of a single object - Integrate and evaluate multiple sources of information in diverse formats and media (e.g., quantitative data, video, multimedia) in order to address a question or solve a problem. | | **Mathematics**   - Reason abstractly and quantitatively - Solve multi-step real-life and mathematical problems posed with positive and negative numbers in any form, using tools strategically. Apply properties of operations to calculate with numbers in any form; convert between forms as appropriate; and assess the reasonableness of answers using mental computation and estimate strategies. - Use variables to represent quantities in real-world or mathematical problem and construct simple equations and inequalities to solve problems by reasoning about the quantities. - Write, read, and evaluate expressions in which letters stand for numbers - Choose a level of accuracy appropriate to limitations on measurement when reporting quantities. - Represent data with plots on the real number line (dots plots, histograms, and boxes). | |

Source: <https://www.nextgenscience.org>
