# Supplementary material for: Building schemas through the lived experiences of an African American roller coaster engineer: Interdisciplinary analyses of students’ dialogues in a physics unit
Source: Front Sociol. 2026 Oct 9;11:1859066. doi: 10.3389/fsoc.2026.1859066 (PMC13690534; doi:10.3389/fsoc.2026.1859066)
Supplement: Supplementary file 2 [file Supplementary_file_2.DOCX]

**Appendix B**

**Positionality Statements of the Researchers**

**Curricula Perspectives and Positioning**

As a Black mother, science education researcher, and curriculum design expert, the first author Quinlan focused on continuously developing curricula expertise which positioned her as the science education program consultant for the new National Geographic Biology textbook that featured diverse scientists, including Black scientists in a biology textbook for the first time (Workman & Quinlan, 2023). This expertise was borne out of a focus on developing science content (Quinlan, 2024a) and supporting multimedia products using pragmatic methodologies (Quinlan, 2021). Quinlan recognizes that STEM and other curricular content ensures reproduction and promotion of White Western cultural knowledge and social capital (Bourdieu, 1977; Painter, 2010) with diminishing returns for learning that positively influences Black children’s identity formation and motivated thinking (Quinlan, 2024b; Quinlan & Farina, 2025). These diminishing returns are evidenced by the continuing challenge for Black students to identify as scientists even with high self-efficacy (Robnett et al., 2015).

This recognition accompanied by the goal to determine *what works*, *how it works,* and *why it works* meant focusing on research which supported the actual development and design of curricula while acknowledging that literature which promotes critical perspectives are also important for advancing this research. The curriculum development used in the treatment and implementation for this research was done only by the first author, Quinlan. Science education researcher Likely was invited to observe and/or participate in its implementation as a community member and researcher, and others were invited to fully participate after the implementation.

**Funds of Knowledge Perspectives and Positioning**

Rollins comes to this research collaboration as a White woman who grew up speaking a Southern Appalachian English dialect in a predominantly-White, working-class NC community. Her family experienced health concerns, loss, alcohol abuse, and trauma related to generational economic hardship. Adults and older siblings taught children innovative "makeshifting" skills, illustrative storytelling, collectivist caregiving and housekeeping, and sustainable farming. Living below the poverty line, she did not experience formal early childhood education. However, she had invaluable educational experiences at home, received a public school education from kindergarten through twelfth grade, and attended a public state university as a first-generation college student with significant help from FAFSA. While public schooling effectively prepared her for careers in social work and early childhood education, her family’s language and literacy practices were viewed as inferior or “wrong” and essentially “schooled out” of them beginning in kindergarten. During graduate studies at a private university in NYC, Moll et. al’s (1992) Funds of Knowledge (FoK) framework helped her further analyze the impact of her families’ language and literacy skills and overall resourcefulness as an early childhood educator.

**Science Education Perspectives and Positioning**

As a Black woman scientist and science educator from the southern United States, Likely’s racial, cultural, and historical identities shape how she engages in science education research. Her experiences across after‑school programs, K–12 classrooms, undergraduate science instruction, laboratory research, and science assessment development continue to reveal the ways Black students are marginalized in science spaces shaped by Whiteness (Sammel, 2009). These lived and professional experiences guide her commitment to creating science learning environments that honor students’ cultural capital rather than requiring assimilation (Bang, et al., 2012; Yosso, 2005).

Grounded in social constructivism, she understands knowledge as co‑constructed and culturally mediated (Nasir, 2011). This perspective aligns with the collaborative, meaning‑making practices embedded in my community and informs how she designs learning experiences and interpret student sensemaking. Her expertise as a researcher, teacher educator, curriculum designer, assessment developer, and maker of hair and body products becomes part of the pedagogical toolkit she leverages when designing culturally sustaining science interventions. These identities are not peripheral; they are foundational to how she collects, understand, and present data in service of Black children who deserve science learning spaces that affirm, honor, and celebrate who they are.

This research, then, became part of ongoing efforts to rethink what equitable science learning can look like when it is intentionally designed *for* Black students and *by* Black people. This project was intentionally a For Us By Us (FUBU) learning experience, drawing from Irizarry’s (2017) framing of education that centers the expertise, histories, and cultural practices of communities marginalized by schooling.

Positioned as researcher, curriculum developer, and facilitator, she brings her lived experience, disciplinary training, and cultural knowledge to this work. While she shares racial and gender identities with the participants in this study, she engaged reflexively, attending to how her presence, interpretations, and commitments shape the research process. Following Probst and Berenson (2014), reflexivity is both a mindset and a set of actions that require ongoing awareness of how she influences what is being studied and how the study, in turn, shapes her.

**Family Engagement Perspectives and Positioning**

As a Black, African American woman, Smith (TaVshea) approaches this research with a focus on family engagement through the lens of the African Diaspora, with her racial and ethnic identity profoundly influencing how she understands educational systems, resource access, and community roles in child development. Her lived experiences as a member of a historically marginalized community inform both her research interests and methodological approaches. Her K-12 public school education shaped her understanding of education as a communal responsibility rather than an isolated institutional endeavor. She embraces a village approach to schooling that recognizes the essential interconnectedness of schools, parents, and community organizations in nurturing children. This perspective stems from witnessing firsthand both the possibilities and limitations of traditional schooling structures, reinforcing my belief that meaningful education emerges from collective investment by multiple partners. Smith, T. is driven by understanding that when parents are empowered with resources, knowledge, and confidence, they become powerful advocates and educators for their children. Families from marginalized communities are too often positioned as deficient when they possess cultural wealth and knowledge that can transform their children's educational experiences.

**Disciplinary Literacy Perspectives and Positionality**

Smith (Delphia) approaches this research as an Afro-Caribbean immigrant woman of color raised in a blue-collared working-class, in what is considered an urban community. As a first-generation college student, her educational journey included both public, private schools and undergraduate study in her home country to graduate school in the United States. These experiences helped to shape the educator she is today. Her disciplinary training in literacy and mixed research has influenced her research focus. She believes that the U.S. is currently in a literacy crisis. Her research centers this area and seeks to improve reading outcomes of U.S. children. Her role in this work is to provide expertise in literacy, specifically disciplinary literacy.
